# On ARGs, pedigrees, and genetic relatedness matrices

**DOI:** 10.1093/genetics/iyaf219

**Published:** 2025-10-08

**Authors:** Brieuc Lehmann, Hanbin Lee, Luke Anderson-Trocmé, Jerome Kelleher, Gregor Gorjanc, Peter L Ralph

**Affiliations:** Department of Statistical Science, University College London, London, WC1E 7HB, United Kingdom; Department of Statistics, University of Michigan, Ann Arbor, MI 48109, United States; Department of Human Genetics, University of Chicago, Chicago, IL 60637, United States; Big Data Institute, Li Ka Shing Centre for Health Information and Discovery, University of Oxford, Oxford, OX3 7LF, United Kingdom; The Roslin Institute and Royal (Dick) School of Veterinary Studies, University of Edinburgh, Edinburgh, EH25 9RG, United Kingdom; Institute of Ecology and Evolution, University of Oregon, Eugene, OR 97402, United States; Department of Data Science, University of Oregon, Eugene, OR 97402, United States

**Keywords:** ancestral recombination graph, genetic relatedness, pedigree relatedness, principal component analysis

## Abstract

Genetic relatedness is a central concept in genetics, underpinning studies of population and quantitative genetics in human, animal, and plant settings. It is typically stored as a genetic relatedness matrix, whose elements are pairwise relatedness values between individuals. This relatedness has been defined in various contexts based on pedigree, genotype, phylogeny, coalescent times, and, recently, ancestral recombination graph. For some downstream applications, including association studies, using ancestral recombination graph-based genetic relatedness matrices has led to better performance relative to the genotype genetic relatedness matrix. However, they present computational challenges due to their inherent quadratic time and space complexity. Here, we first discuss the different definitions of relatedness in a unifying context, making use of the additive model of a quantitative trait to provide a definition of “branch relatedness” and the corresponding “branch genetic relatedness matrix”. We explore the relationship between branch relatedness and pedigree relatedness (i.e. kinship) through a case study of French–Canadian individuals that have a known pedigree. Through the tree sequence encoding of an ancestral recombination graph, we then derive an efficient algorithm for computing products between the branch genetic relatedness matrix and a general vector, without explicitly forming the branch genetic relatedness matrix. This algorithm leverages the sparse encoding of genomes with the tree sequence and hence enables large-scale computations with the branch genetic relatedness matrix. We demonstrate the power of this algorithm by developing a randomized principal components algorithm for tree sequences that easily scales to millions of genomes. All algorithms are implemented in the open source tskit Python package. Taken together, this work consolidates the different notions of relatedness as branch relatedness and, by leveraging the tree sequence encoding of an ancestral recombination graph, provides efficient algorithms that enable computations with the branch genetic relatedness matrix that scale to mega-scale genomic datasets.

## Introduction

In its most general sense, genetic relatedness refers to the notion of similarity between individuals’ genomes. These similarities are usually summarized as a pairwise comparison of the genomes within an individual and between individuals, or groups of individuals. In the literature, these definitions and estimators are referred to by a wide variety of terms: kinship, coancestry, inbreeding, etc.; in this we paper use “relatedness” (and provide explicit definitions). As a central concept in genetics, relatedness is used in many applications (Weir et al. 2006 ; Speed and Balding 2015). For example, measures of relatedness have been used to describe genetic variation within and between individuals and groups of individuals in population genetics (Crow and Kimura 2009, Chapter 4), to analyze phenotype covariation between close and distant relatives in quantitative genetics (Falconer and Mackay 1996, Chapter 9), and to estimate genetic changes in phenotypic variation over time in evolutionary genetics (Arnold 2023, Chapter 5). For a set of individuals, it is helpful to store their pairwise relatedness values in a *genetic relatedness matrix*, often abbreviated *GRM* (also known by other terms as well, including the “numerator relationship matrix”). Over time, genetic relatedness and GRMs have been defined according to pedigree (Fisher 1919 ; Wright 1922 ), genotype (Cotterman 1940; Malécot 1948 , 1969 ; VanRaden 2008), phylogeny (Felsenstein 1985; Lynch 1991), coalescent times (Slatkin 1991), and recently, ancestral recombination graphs (Fan et al. 2022 ; Tsambos 2022; Zhang et al. 2023 ; Tang and Chiang 2025).

Ancestral recombination graphs (ARGs) describe the network of inheritance relations between a set of individuals via the action of recombination and mutation within a (usually implicit) pedigree (Brandt et al. 2024 ; Lewanski et al. 2024; Nielsen et al. 2024; Wong et al. 2024), and so provide a common framework in which to consider the various concepts of relatedness. Although ARGs are not directly observable, there has been significant recent progress in inferring ARGs from a sample of DNA sequences (Rasmussen et al. 2014; Kelleher et al. 2019; Speidel et al. 2019; Zhang et al. 2023; Deng et al. 2024; Gunnarsson et al. 2024). This has been accompanied by computational advances that enable the highly efficient storage and processing of ARGs (Kelleher et al. 2016 ; DeHaas et al. 2024; Zhu et al. 2024). ARGs can store genetic data in a way that is not only more compact but also more expressive, as they provide direct access to haplotypes, local trees, and estimated dates for inferred recombination, mutation, and coalescence events. In this paper we use the *succinct tree sequence* ARG encoding (Ralph et al. 2020; Wong et al. 2024) available through the tskit library.

In addition to providing a unifying framework, ARGs have led to new formulations of relatedness. The “eGRM” of Fan et al. (2022) defines the relatedness between two individuals in terms of the total area of branches in the ARG that are ancestral to both, similar to previous single-tree definitions (Slatkin 1991). Fan et al. (2022) showed this is the expected genotype relatedness under a Poisson model of mutation, a special case of a more general duality between “branch” and “site” statistics (Ralph 2019; Ralph et al. 2020). The same concept was used by Zhang et al. (2023), although with different terminology, who connected their definition of the “ARG-GRM” to the time to most recent common ancestor (TMRCA) of a single tree (Slatkin 1991; McVean 2009). There are now many different notions of relatedness (see Box 1 for a brief overview), usually defined as an expectation of some quantity (e.g. pedigree relatedness is the expected genetic similarity within a pedigree). We therefore use the more precise terms “branch relatedness” and “branch GRM” rather than previously proposed “eGRM” or “ARG-GRM” to avoid confusion.

Box 1: **A Brief History of Genetic Relatedness**

Genetic relatedness was first formalized by Wright (1922), who introduced the coefficients of relationship and inbreeding in the context of a pedigree based on the path (correlation) analysis of phenotypic values for pedigree members. He also showed how to compute these coefficients in a general pedigree by tracing all pedigree lineages between relatives. Emik and Terrill (1949) and Cruden (1949) devised a simpler procedure to compute these coefficients between all pairs of pedigree members, which was later formalized as a recursive algorithm (Henderson 1976). The algorithm fills in a symmetric genetic relatedness matrix (GRM), which is the key object for statistical genetics, particularly through its use in linear mixed models (Falconer and Mackay 1996; Henderson 1984, Lynch and Walsh (1998, Chapter 26), Mrode and Pocrnic (2023, Chapter 3)). However, pedigree information is limited because it can only quantify the relatedness relative to pedigree founders (Wright 1965; Jacquard 1975), pedigrees are increasingly incomplete backwards in time (e.g. Legarra et al. 2015), and pedigrees measure expected rather than realized relatedness due to recombination and mutation (e.g. Hill and Weir 2011; Thompson 2013; García-Cortés et al. 2013).

Relatedness was later redefined with respect to genotypes using concepts of identity by descent (IBD) and identity by state (IBS) by Cotterman (1940) and Malécot (1948, 1969), before DNA data was readily available. Early developments of molecular genetics enabled generation of DNA data and estimation of genotype-based relatedness (see review by Weir et al. 2006). As anticipated by Thompson (1975), early studies found that the number of markers is critical for the accuracy of estimates and distinguishing different types of relationships for pairs of individuals. Increasing the number of markers improved the accuracy, but also revealed substantial variation from the expected pedigree relatedness between pairs of individuals - overall as well as along genome regions - due to recombination (Weir et al. 2006).

Further developments in molecular genetics streamlined genome-wide genotyping with SNP arrays and increasingly whole-genome resequencing such that today we have genotype datasets with thousands to millions of individuals (e.g. Turnbull et al. 2018b; Bycroft et al. 2018; Ros-Freixedes et al. 2022b). This data-abundance has reinvigorated population genetics studies of variation within and between populations (Begun et al. 2007; Langley et al. 2012), and statistical genetics studies of complex trait architecture (Burton et al. 2007; Abdellaoui et al. 2023) and prediction of such traits (Meuwissen et al. 2001, 2013). Depending on the aims of the study (Speed and Balding 2015), we now use a number of different relatedness estimators (e.g. VanRaden 2008; Yang et al. 2010; Manichaikul et al. 2010; Speed et al. 2012; Weir and Goudet 2017, 2018; Ochoa and Storey 2021; Mary-Huard and Balding 2023). Variants of the genotype sample covariance between individuals, known as the genomic relationship/relatedness matrix (GRM), are commonly used (VanRaden 2008; Yang et al. 2010; Speed et al. 2012). These estimators largely treat loci independently, though some approaches leverage linkage between loci to improve delineation between IBS and IBD relatedness (e.g. Visscher et al. 2006 ; Browning and Browning 2012; Thompson 2013; Hickey et al. 2013 ; Edwards 2015 ; Pook et al. 2019 ; Nait Saada et al. 2020). The ease of use is the primary reason for treating genotype loci independently. Conversely, to leverage linkage between loci, one needs to phase genotypes, operationally define similarity between the resulting haplotypes, and then estimate relatedness based on these haplotype similarities.

There is a growing interest to use ancestral recombination graphs (ARG) as the ultimate description of such haplotype similarities for a sample of individuals. ARGs are therefore enabling the study of relatedness and its use in downstream genetic analyses (Fan et al. 2022; Tsambos 2022; Zhang et al. 2023; Link et al. 2023; Schraiber et al. 2024). Relatedness based on an ARG leverages allele differences between individuals, but also linkage between these alleles, hence connecting IBS and IBD concepts, capturing typed and untyped loci, and time-varying changes in population structure due to ancient and recent demographic events (Fan et al. 2022; Young 2022; Zhang et al. 2023; Harris 2023).

Recent applications of these methods have highlighted the advantages of using branch information to improve genetic analyses. Fan et al. (2022) demonstrate that the branch GRM (their eGRM) better describes population structure relative to the corresponding genotype GRM, even when based on the same genetic information, and can provide time-resolved characterizations of population structure by considering shared branch areas on particular subsets of the ARG defined by specific time intervals. Tang and Chiang (2025) modified the “eGRM” to better reveal the recent relatedness among admixed individuals. Tsambos (2022) developed a method to find DNA segments that are identical-by-descent (IBD) for pairs of individuals in a given ARG and then summarize these outputs, possibly as an “IBD GRM”, which provides an ARG-based analog to the pedigree GRM. Link et al. (2023) applied a branch GRM to improve mapping of quantitative trait loci in the presence of allelic heterogeneity and in understudied populations. Zhang et al. (2023) use a branch GRM (their ARG-GRM) to estimate heritability and to perform a “genealogy-wide association scan”, showing that this approach can be more powerful at detecting the effect of rare variants than association analysis on SNP array genotypes imputed to whole-genome sequence genotypes. Gunnarsson et al. (2024) extended this work to a large whole-genome sequence dataset and Zhu et al. (2024) used randomized linear algebra to scale the estimation of heritability and region-based association testing with branch GRM.

This landscape of work presents a confusing array of types of “relatedness”, that differ in both subtle and fundamental ways. Which type of “relatedness” is best suited for a given task depends both on the data used to estimate relatedness and on the task at hand. For instance, the branch GRM measures sharing of genetic material represented in the ARG that was used to compute it, while the pedigree GRM, since it averages over transmission of genetic material within the pedigree, might better measure sharing of genetic material across a wider genomic region. This is similar to the fact that the genotype GRM measures sharing of precisely those genetic variants that are used to compute it, while the branch GRM might better measure sharing of unobserved genetic variants. If one is confident that the sequenced portions of the genome are those relevant for the task at hand, then a GRM based on observed data (genotype or branch) might be best. Indeed, if one knows which portions of the genome are likely to harbor variation for a given trait, and that all causal variation is represented in the sequencing data, it might be best to use the genotype GRM constructed from only those regions. On the other hand, if one has constructed a pedigree based on only a relatively small number of loci, then the pedigree GRM might provide a better estimate of what the branch or genotype GRM would be given full genetic data. Below, we discuss some relationships and differences between some of these types of relatedness.

In addition to the choice of relatedness measure, the algorithm used to compute it plays an important role in determining the computational feasibility of applying these metrics to large datasets. The scalability of current exact ARG-based relatedness methods is constrained by their need to generate and store the full branch GRM. As the GRM encodes all pairwise relationships among *n* samples, it requires at least $O(n^{2})$ time and space to compute. Several currently available datasets of core interest for these methods consist of hundreds of thousands of samples (Caulfield et al. 2017; Bycroft et al. 2018; Turnbull et al. 2018a; Backman et al. 2021; Halldorsson et al. 2022; Ros-Freixedes et al. 2022b; UK Biobank Whole-Genome Sequencing Consortium et al. 2023; All of Us Research Program Genomics Investigators et al. 2024), and genomic datasets with millions of samples are increasingly available (e.g. Cesarani et al. 2022; Stark et al. 2024; Cole et al. 2025; Cook et al. 2025). At this scale, algorithms with quadratic time and space complexity are simply not feasible. However, the GRM itself is often not the goal; rather, we are usually interested in what we can do *with* the GRM. For example, population genetic applications such as principal component analysis (PCA) and quantitative genetic applications such as estimation of heritability, are defined in terms of core linear algebra operations performed with the GRM, and the outputs are of much smaller dimension. Given that all the information in a GRM is encoded in an ARG, there is the possibility that we can bypass generating large intermediate matrices and instead compute the quantities of interest directly. This approach was used by Zhu et al. (2024), who use the ARG for fast, approximate GRM-vector multiplication. Indeed, the ARG can be seen as a sparse matrix representation of the genotype matrix which can hence naturally be used for efficient computation (Ralph et al. 2020).

In this paper, we begin by defining a trait-centric concept of genetic relatedness, following long-standing approaches in the field (Fisher 1919; Wright 1922). We show how branch relatedness arises as the covariance of a trait determined additively along the branches of an ARG, and how this relates to other measures of relatedness. We then illustrate these definitions and the relationships between pedigree relatedness and branch relatedness using simulated data from a real pedigree of French–Canadian individuals. Next, we describe a relatively efficient algorithm to compute the entire branch GRM that has complexity $O(tn^{2})$, where *t* is the number of local trees (or equivalently, number of recombination breakpoints) in an ARG. As discussed in the previous paragraph, explicit representations of the entire GRM are necessarily limited in scale, so we turn to matrix-vector products. We then present an algorithm to compute the product of the branch GRM with an arbitrary vector, and show that it has O(n+tlogn) time complexity and O(n) space complexity. We can therefore compute branch GRM-vector products substantially faster and with less memory than the branch GRM itself. We illustrate the utility of this approach by presenting a randomized singular value decomposition method for PCA of the branch GRM (implemented in tskit), and show that it scales to millions of samples via benchmarks. In this paper we focus on the branch GRM, whose utility is still being explored but has been demonstrated for trait prediction, fine mapping, and related tasks (Fan et al. 2022; Link et al. 2023; Gunnarsson et al. 2024; Zhu et al. 2024); however, similar algorithms can be used for the genotype GRM.

## Results

### ARGs and tree sequences

We first introduce our notation for ARGs, following Kelleher et al. (2016, 2018), and Wong et al. (2024). Using this terminology, an ARG represents the history of a set of sampled genomes by a collection of *nodes* and *edges*. Each chromosome of an individual is represented by a *node*, and each node has an associated *time*, indicating when the individual was born. In diploids, the two haploid genomes of a genotyped individual are represented by two *sample* nodes. *Ancestral* nodes represent genomes of non-genotyped individuals. Each branch encodes the inheritance of some genome segment by some “child” node from a “parent” node (despite the terminology, these two may be separated by more than one generation). The *span* of an edge is the length of the inherited segment of genome, and the *length* of a branch is the number of generations across which the segment was inherited, that is, the difference between the times of parent and child nodes. So, a given edge may represent branches in many local trees: so, we (mostly) use “edge” to refer to the component of the ARG that implies inheritence of a segment of genome, and “branch” to refer to the part of a single local tree. Genetic variation is represented in this structure by recording where in the ARG mutations occurred. For example, if we say that a mutation that produces a C nucleotide occurs at genomic position *x* on the edge from some parent *p* to a child *c*, then (1) the mutation has occurred somewhere in the chain of inheritances by which *c* has inherited the genetic material from *p*; and (2) any other nodes that inherit from *c* at position *x* will carry a C, unless another mutation intervenes. Finally, recombination events are implicitly encoded by edges between child and parent nodes, that is, a child node can inherit from different nodes of different parents. Inheritance relationships at each location of the genome are described by a *local tree*, and subsequent local trees are separated by the genomic locations of recombination events. Branches are often repeated across many adjacent trees, reflecting common genealogical relationships (i.e. individual *c* inherits from individual *p*) that are shared across relatively long sections of the genome.

The *succinct tree sequence*, or *tree sequence* for short, is an efficient ARG encoding (Kelleher et al. 2016, 2018; Wong et al. 2024). The data structure is based on a succinct description of nodes, edges, and mutations as described above, and can be used to efficiently recover and process the sequence of local trees that describe how the samples are related on each consecutive section of the chromosome. Branches that are shared across multiple adjacent trees are encoded as a single edge in the data structure. This property enables the implementation of efficient computational algorithms.

### A trait-centric notion of genetic relatedness

We now discuss how genetic relatedness can be characterized as the covariance of a hypothetical trait between individuals. The definitions are not new, but are often not found explicitly in the literature. The model begins in essentially the same place as the infinitesimal model as described in Barton et al. (2017), but with patterns of inheritance known instead of averaged over. This conceptual exercise will serve to clarify the relationship between distinct definitions of relatedness based on the ARG.

Consider an additive trait, that is, a trait whose genetic value is the sum of effects associated with each allele carried by an individual. We write Z(i) for the genetic value of the trait for individual *i*. Suppose that the genotypes at each locus are from some alphabet A, and that at each locus ℓ in the genome there is an “ancestral” allele $a_{\ell }$. The additive effect of allele *x* at locus ℓ is $Z_{\ell ,x}$, which is relative to the ancestral allele, so that $Z_{\ell ,a_{\ell }}=0$. More discussion of this choice can be found in section Treatment of reference alleles of the Appendix. Then, an individual’s genetic value is the sum of the effects of alleles across all $n_{L}$ loci, averaged across genome copies. We will write $G_{i,\ell ,g}$ for the allele of the $g^{\mathrm{th}}$ genome copy of individual *i* at locus ℓ, so that a *p*-ploid individual *i* has genetic value:


$$Z(i)=\frac{1}{p}\sum_{g=1}^{p}\sum_{\ell =1}^{n_{L}}Z_{\ell ,G_{i,\ell ,g}}.$$

Finally, suppose that the effects of each non-ancestral allele $Z_{\ell ,x}$ are independently drawn from a probability distribution with mean zero and variance $\sigma^{2}$. The choice to average across the *p* genome copies (as opposed to, say, sum them) is only consequential for situations with mixed ploidy and could be thought of as implying a particular model of dosage compensation. Mixed ploidy arises with sex chromosomes or haplodiploids (Grossman and Eisen 1989), or summarizing relatedness between groups with different number of individuals (Cockerham 1967). Many measures of relatedness make use of this trait model (explictly or implictly), in which case relatedness is proportional to covariance between individuals’ trait values. We now demonstrate this equivalence.

For simplicity, suppose for the moment that all loci are bi-allelic, so $G_{i,\ell ,g}\in \{0,1\}$, and $Z_{\ell ,0}=0$. See section Multi-allelic loci in haploids in the Appendix for a more general discussion. Under this model, we write p(i,ℓ) as the proportion of alleles carried by individual *i* at locus ℓ that are not ancestral (so $p(i,\ell )=(G_{i,\ell ,1}+G_{i,\ell ,2})/2$ for diploids). Suppose we have $n_{I}$ haploid individuals $1,\ldots ,n_{I}$ with genetic values $Z(1),\ldots ,Z(n_{I})$. We write $\bar{Z}=\frac{1}{n_{I}}\sum_{i=1}^{n_{I}}Z(i)$ to be the sample mean and define the mean allele frequency among these individuals as $\bar{\;p}(\ell )=(p(1,\ell )+\cdots +p(n_{I},\ell ))/n_{I}$. The covariance of the traits after centering to the sample mean is:


(1) $$\begin{array}{rl} & C\text{ov}\left[Z(i)-\bar{Z},Z(\;j)-\bar{Z}\right]\\ & \;=\sigma^{2}\sum_{\ell =1}^{n_{L}}\left(\;p(i,\ell )-\bar{\;p}(\ell )\right)\left(\;p(\;j,\ell )-\bar{\;p}(\ell )\right). \end{array}$$

Note that this is the covariance of Z(i) and Z(j) as random variables, averaging over random assignment of allelic effects, but with genotypes fixed (p(i,ℓ) is not random). The centering to the sample mean ensures that loci with no variation do not contribute to the covariance expression (1), because at these loci $p(i,\ell )=\bar{\;p}(\ell )$. See section Covariance between uncentered traits in the Appendix for further discussion on centering.

We now derive an alternative form of expression (1) to highlight a connection to the familiar genotype GRM. If *U* and *V* are random, uniformly chosen individuals from the sample, and *L* a random, uniformly chosen locus, then, we can rewrite $\bar{Z}=E[Z(U)]$ and $\bar{p}(\ell )=E[p(U,\ell )]$. Consequently, the two sides of (1) are also equal to:


(2) $$\begin{array}{rl} & E\left[(Z(i)-Z(U))(Z(\;j)-Z(V)\right)]\\ & \;=\sigma^{2}E\left[\left(\;p(i,L)-p(U,L)\right)\left(\;p(\;j,L)-p(V,L)\right)\right], \end{array}$$

where the expectation is averaging over choice of *U*, *V*, and *L*.

To see the connection to the genotype GRM in haploids, we can treat $G\in \{0,1{\}}^{n_{I}\times n_{L}}$ as the genotype matrix for $n_{I}$ haploid individuals at $n_{L}$ loci. We are interested in the covariance between individuals *i* and *j*, that is, between the two genomes in rows *i* and *j* of G. Let $G^{c}$ be the *column-centered* haplotype matrix with entries $G_{i,\ell }^{c}=G_{i,\ell }-\bar{\;p}(\ell )$. A common definition of covariance is:


$$C=\frac{1}{n_{L}}G^{c}{G^{c}}^{⊺},$$

so that the covariance between individuals *i* and *j* based on their genotypes is:


(3) $$C_{i,j}=\frac{1}{n_{L}}\sum_{\ell =1}^{n_{L}}(G_{i,\ell }-\bar{\;p}(\ell ))(G_{\;j,\ell }-\bar{\;p}(\ell )).$$

This expression (3) is the kernel of many variants of genotype GRM (VanRaden 2008; Yang et al. 2010; Speed and Balding 2015; Zhang et al. 2023), apart from difference between the haploid and diploid setting, with the latter being an aggregate form of the former (Cockerham 1967; Smith and Allaire 1985). This expression (3) is also equal to (1) divided by $n_{L}$, after setting $\sigma^{2}=1$. The corresponding expression for diploids uses in place of G the allele dosage matrix whose entries are the proportion of non-reference alleles carried by the individual. It is more common in the literature to define the allele dosage matrix as the *number* of non-reference alleles; here we define it as the proportion so that it agrees with (1); this is necessary because of the convention to define Z(i) as the average across the *p* genome copies. For diploids this results in an additional factor of four.

Many definitions of relatedness weight the contribution of the $\ell^{\mathrm{th}}$ locus by $(\bar{p}(\ell )(1-\bar{\;p}(\ell )){)}^{\alpha }$. We take α=0 for simplicity, but the discussion below applies more generally.

A third interpretation of this covariance can be derived as follows. As before, take *i* and *j* as two fixed haploid individuals, and define the random variable $\left(X_{i},X_{j},X_{U},X_{V}\right)$ to be $\left(G_{i,L},G_{\;j,L},G_{U,L},G_{V,L}\right)$, where *U*, *V*, and *L* are defined as above. In other words, $\left(X_{i},X_{j},X_{U},X_{V}\right)$ is the alleles of those four individuals (*i*, *j*, and then two chosen at random, with replacement) at a uniformly chosen locus. Then, as shown in section Proof of equation (4) of the Appendix, it turns out that:


(4) $$\begin{array}{rl} C_{i,j} & =\frac{1}{2}\left(P(X_{i}=X_{j})-P(X_{i}=X_{U}\right)\\ & \;-P(X_{j}=X_{V})+P(X_{U}=X_{V})). \end{array}$$

This expression is more readily extendable to multi-allelic data.

We therefore have the following three equivalences (1), (3) and (4):


$$\begin{array}{rl} C_{i,j} & =\frac{1}{n_{L}\sigma^{2}}C\text{ov}\left[Z(i)-\bar{Z},Z(\;j)-\bar{Z}\right]\;(1)\\ & =\frac{1}{n_{L}}\sum_{\ell =1}^{n_{L}}(G_{i,\ell }-p_{\ell })(G_{j,\ell }-p_{\ell })\;(3)\\ & =\frac{1}{2}\left(P(X_{i}=X_{j})-P(X_{i}=X_{U}\right)\\ & \;-P(X_{j}=X_{V})+P(X_{U}=X_{V})).\;(4) \end{array}$$

From the third equivalence (4), the quantity $n_{L}C_{i,j}$ has the following interpretation. Let m(i,j) denote the number of pairwise allele matches between the individual *i* and *j*, and let *U* and *V* be independently chosen individuals from the set of individuals. Then the quantity $n_{L}C_{i,j}$ is the expected number of pairwise allele matches between *i* and *j* relative to the rest of individuals:


(5) $$n_{L}C_{ij}=E[m(i,j)-m(i,U)-m(\;j,V)+m(U,V)],$$

where the expectation is over the choice of U and V. This interpretation is closely related to the definition of kinship between individuals *i* and *j* as the “the probability of a match between alleles drawn at random from each of them”, averaged over loci, and with the alleles drawn with replacement if i=j (Malécot 1948, 1969; Speed and Balding 2015). See also Weir and Goudet (2017, 2018) and Ochoa and Storey (2021) on other “relative” kinship estimators.

### A trait-centric perspective on the branch relatedness

We now describe a closely related notion of branch relatedness. Suppose that we only observe the relationships in the ARG, not the mutations that appear in it. This is similar to the starting point of pedigree relatedness, but we assume we also know full ancestry of each genome all the way to the roots of each local tree (the MRCAs) and which portions of the genomes were inherited in each relationship. The expected number of mutations that appear on a segment of genome of *s* base pairs inherited across *b* generations is proportional to b×s. In other words, the expected number of mutations on an edge *e* of length $b_{e}$ and span $s_{e}$ is proportional to its area, $A_{e}=b_{e}\times s_{e}$. If the effect of each mutation has variance $\sigma^{2}$ and these are additive, then the variance of the edge effect is $A_{e}\sigma^{2}$. This is because the variance of the sum of a random number *N* of independent and identically distributed mean-zero terms is the mean of *N* multiplied by the variance of the terms.

Now, let us define this situation mathematically, analogously to the previous section. Let $S_{i,e}=1$ if sample *i* is a descendant of edge *e* and $S_{i,e}=0$ otherwise, $Z_{e,1}$ be the effect of edge *e*, and $Z_{e,0}=0$ (as before). Then the trait for individual *i* has genetic value:


$$Z(i)=\sum_{e}Z_{e,S_{i,e}},$$

where the sum is over edges in the ARG. As before, suppose that the edge effects $Z_{e,1}$ are independent mean-zero random variables with variance $A_{e}\sigma^{2}$. Then, we define *branch relatedness* to be the covariance of the trait values, after centering to the sample mean:


(6) $$B_{i,j}=C\text{ov}\left[Z(i)-\bar{Z},Z(\;j)-\bar{Z}\right],$$

with $\bar{Z}$ defined as before. This is similar to (1), but differs in that here the covariance averages not only over allelic effects, but also over location of the mutations, so E[C]=B if the expectation averages over the mutations that define C under the infinite-sites model. This is an example of a general relationship described in Ralph et al. (2020). Since covariance between the traits of *i* and *j* derives from sharing of edge effects, denoting A(i,j) to be the total area of edges ancestral to individuals *i* and *j*, and with randomly chosen individuals *U* and *V* as above, it turns out that:


(7) $$B_{i,j}=E[A(i,j)-A(i,U)-A(\;j,V)+A(U,V)],$$

where the expectation is over the choice of *U* and *V*. See Table 1 for a summary of branch and genotype relatedness definitions. The branch relatedness can also be rewritten as a weighted average of coalescence times, as noted by McVean (2009), Fan et al. (2022) and Zhang et al. (2023). Let $s_{k}$ be the genome sequence length corresponding to the $k^{\mathrm{th}}$ local tree, and within this tree define b(i,j,k) be the total length of branches ancestral to both haploid individuals *i* and *j*, let t(i,j,k) be the TMRCA of *i* and *j*, and let $\hat{t}(k)$ the time of the root. Supposing that *i* and *j* are both at time 0, then the time of the root is equal to the TMRCA plus any additional, shared, branch lengths:


(8) $$\hat{t}(k)=b(i,j,k)+t(i,j,k).$$

We can use this relationship to split (6) by local tree as follows, using the fact that $A(i,j)=\sum_{k}s_{k}b(i,j,k)$ in (7):


$$\begin{array}{rl} B_{i,j} & =\sum_{k=1}^{n_{T}}s_{k}E[b(i,j,k)-b(i,U,k)-b(\;j,V,k)+b(U,V,k)]\\ & =\sum_{k=1}^{n_{T}}s_{k}E[t(i,U,k)+t(\;j,V,k)-t(i,j,k)-t(U,V,k)], \end{array}$$

where $n_{T}$ is the number of local trees in the ARG, and the expectation averages over *U* and *V*.

**Table 1.** Summary of relatedness definitions.

Type of Relatedness	Interpretation	Definition	Notation
**Genotype**	Covariance of genetic value of hypothetical trait with effects for each allele.	$\begin{array}{rl} C_{i,j} & =C\text{ov}[Z(i)-\bar{Z},Z(\;j)-\bar{Z}]\\ Z(i) & =\sum_{\ell =1}^{n_{L}}Z_{\ell ,G_{i,l}}\\ Z_{\ell ,0} & \equiv 0\\ E[Z_{\ell ,1}] & =0,\;V\text{ar}(Z_{\ell ,1})\propto 1 \end{array}$	- Z(i): value of hypothetical trait in individual *i*
			- $\bar{Z}$: mean genetic value in sample
			- $Z_{\ell ,G_{i,l}}$: allele effect at locus ℓ in individual *i*
			- $G_{i,\ell }$: allele at locus ℓ in individual *i*
			- $n_{L}$: number of loci
**Genotype**	Expected number of pairwise allele matches between pair of individuals relative to the rest of individuals	$\begin{array}{rl} C_{i,j} & =\frac{1}{n_{L}}E[m(i,j)\\ & \;-m(i,U)\\ & \;-m(\;j,V)\\ & \;+m(U,V)] \end{array}$	- m(i,j): number of pairwise allele matches between individuals *i* and *j*
			- *U*, *V*: randomly selected individuals in sample
			- $n_{L}$: number of loci
**Branch**	Covariance of genetic value of hypothetical trait with effects for each branch.	$\begin{array}{rl} B_{i,j} & =C\text{ov}[Z(i)-\bar{Z},\\ & \;Z(\;j)-\bar{Z}]\\ Z(i) & =\sum_{e=1}^{n_{E}}Z_{e,S_{i,e}}\\ Z_{e,0} & \equiv 0\\ E[Z_{e,1}] & =0,\;V\text{ar}(Z_{e,1})\propto A_{e} \end{array}$	- $S_{i,e}$: indicator denoting whether individual *i* is a descendant of branch *e*
			-$n_{E}$: number of branches in the ancestral recombination graph
			- $A_{e}$: area of branch *e*
**Branch**	Expected shared branch area between pair of individuals relative to the rest of the individuals	$\begin{array}{rl} B_{i,j} & =E[A(i,j)\\ & \;-A(i,U)\\ & \;-A(\;j,V)\\ & \;+A(U,V)] \end{array}$	- A(i,j): total area of branches ancestral to both *i* and *j*
			- U,V: randomly selected individuals in sample
**Pedigree**	Probability that a pair of homologous alleles, drawn from individuals *i* and *j*, are identical-by-descent.	$\theta_{i,j}=\sum_{a\in A_{i,j}}\frac{1+f^{a}}{2^{g_{a}+1}}$	- $A_{i,j}$: set of most recent common ancestors of *i* and *j*
			- $f_{a}$: inbreeding coefficient of ancestor *a*
			- $g_{a}$ number of parent-child links in the lineage path linking *i* and *j* via *a*

Our definition differs slightly from the eGRM by Fan et al. (2022). Let $S_{i,e,k}=1$ if sample *i* is a descendant of edge *e* in the $k^{\mathrm{th}}$ tree and $S_{i,e,k}=0$ otherwise, and ${\bar{S}}_{e,k}=\sum_{i=1}^{n_{I}}S_{i,e,k}/n_{I}$ for the proportion of samples inheriting from *e*. Also, write $b_{e}$ for the length of *e*. Then (6) can be rewritten as:


$$B_{i,j}=\sum_{k=1}^{n_{T}}s_{k}\sum_{e\in T_{k}}b_{e}(S_{i,e,k}-\bar{S}(e,k))(S_{\;j,e,k}-\bar{S}(e,k)),$$

where the second sum is over branches *e* in the $k^{\mathrm{th}}$ tree. On the other hand, Fan et al. (2022) define the eGRM as:


(9) $$eGRM_{i,j}=\frac{1}{A_{T}}\sum_{k=1}^{n_{T}}s_{k}\sum_{e\in T_{k}}b_{e}\frac{\left(S_{i,e,k}-\bar{S}(e,k))(S_{\;j,e,k}-\bar{S}(e,k)\right)}{\bar{S}(e,k)(1-\bar{S}(e,k))},$$

where $A_{T}=\sum_{k}\sum_{e\in T_{k}}s_{k}b_{e}$ is the total area of the ARG. The different denominator normalizes the contribution of each edge according to its standard deviation in the population, and is equivalent to the $\sqrt{2p(1-p)}$ standardization often used in the genotype GRM.

Relatedness and divergence are closely related, as demonstrated by the relationship (8). Let d(i,j,k) be the distance in the $k^{\mathrm{th}}$ tree between *i* and *j*, and $r_{k}$ the root of the $k^{\mathrm{th}}$ tree. A more general relationship that does not assume *i* and *j* are both at time zero is (Semple and Steel 2003, Chapter 7):


(10) $$d(i,r_{k},k)+d(\;j,r_{k},k)=2b(i,j,k)+d(i,j,k).$$

That is, the sum of the distances from each to the root is equal to the distance between them plus twice the distance from their MRCA to the root. Let R(i) denote the sum along the genome of the distances from *i* to the root and D(i,j) the sum along the genome of the distances between *i* and *j* in the local trees. Then D(i,j) is the unnormalized branch genetic divergence between *i* and *j* (Ralph et al. 2020), and summing the previous relation across the genome, we get:


(11) R(i)+R(j)=2A(i,j)+D(i,j).

Rearranging and substituting into the expression for branch relatedness (6), centering cancels the terms with *R*, giving:


(12) $$B_{i,j}=-\frac{1}{2}E[D(i,j)-D(i,U)-D(\;j,V)+D(U,V)].$$

Thought of as matrices, if $P=I-11^{⊺}/n_{I}$ is the $n_{I}\times n_{I}$ centering matrix, the above equation says that B=−PDP/2. For more discussion and other relationships between relatedness and divergence, see Zhang et al. (2023), Supplementary Note 3.

### Branch PCA

Principal component analysis (PCA) is a commonly used technique to quantify and visualize population structure from genotype data. Mathematically, PCA projects samples onto a set of orthogonal axes, each defined as a linear combination of genotype values across SNPs or other genetic variants. An iterative characterization of PCA is as follows: choose the first principal component to be the axis that captures the maximum possible variance in the data, then choose the second principal component that maximizes variance whilst being orthogonal to the first, and so on. The first three or four principal components are often presented as a low-dimensional summary of population structure.

Principal components can be found, equivalently, as eigenvectors of the (centered) genotype GRM or singular vectors of the underlying (centered) genotype matrix. Both decompositions can be efficiently approximated with randomized algorithms that can operate on matrices only implicitly defined through matrix-vector products (Halko et al. 2011). McVean (2009) gave a genealogical interpretation of PCA, while Fan et al. (2022) showed that branch PCA can in some cases better capture recent population structure than genotype PCA, even when based on the same genotype information. In section Computation we describe an efficient algorithm for such a product that bypasses the construction of branch GRM and operates directly on tree sequence encoding of an ARG. However, we first discuss the connection between pedigree and branch relatedness.

### Connection between pedigree and branch relatedness

There are various measures of relatedness within a given pedigree. The most common of these is the “kinship coefficient” (or “coefficient of coancestry”), defined for two individuals as the probability that a randomly chosen one of each of their two homologous copies inherit from the same ancestral genome within the pedigree at a given locus, that is, that they are identical by descent (IBD) within the pedigree (Malécot 1969, Section 1.3). This probability is computed averaging over segregation of genetic material within the pedigree; if we took the patterns of genetic inheritance as given, we would arrive at the analogous “IBD GRM” discussed by Tsambos (2022). We refer to this as “pedigree kinship”, although the term “pedigree relatedness” is also used.

While pedigree kinship and branch relatedness seem similar, and in some situations pedigree kinship can provide a good approximation to branch or genetic relatedness, they differ fundamentally in what they measure. While pedigree (or IBD) kinship measures the probability of identity with reference to a particular time (or set of ancestors) in the past, branch relatedness measures shared branch area. This means that the two differ in units. The natural units of pedigree kinship is in genomic distance, since it gives the proportion of the genome on which the two coalesce within the pedigree. On the other hand, branch relatedness is in units of genomic distance multiplied by time, since it is shared area. One way to see this is to note that pedigree kinship is derived ignoring the contribution of any new mutations within the pedigree, while branch relatedness explicitly counts the opportunity for new mutations (and hence introduces a factor of “time” to the units). What about genetic relatedness, $C_{i,j}$? Because genetic relatedness $C_{i,j}$ can be written using probabilities of identity (equation (4)), it seems analogous to IBD kinship within a pedigree or an ARG, but the relationship $E[C_{i,j}]=B_{i,j}$ suggests that it is better to think about (mutation/site-based) genetic relatedness as having units of genome × time as well.

Despite this distinction, there is a close relationship between branch relatedness and pedigree kinship, deriving from the oft-used intuition that most deviations from average relatedness comes from sharing of close relatives. Here, we provide a short and informal derivation. First, note that the branch relatedness between two haploid genomes can be decomposed into two parts: where their MRCA is within the pedigree, and where it is not, and that typically the pedigree is short, so contributions to shared branches from within the pedigree can be ignored. Pedigree kinship $\theta_{i,j}$ between a pair of individuals is the expected proportion of the genome on which the two inherit from a common ancestral genome within the pedigree, and so gives the expected proportion of the genome where the MRCA is within the pedigree. Where the MRCA is not within the pedigree, the length of shared branches above the two is equal to the length of shared branches above the two founders from which they inherit; call this, averaged over distinct founders, *A*. On the other hand, if the two share a MRCA within the pedigree, then they share the branch above that MRCA; call this, again averaged over distinct founders, *R*. Putting this together, we have that the shared branch area above two individuals is roughly $\theta_{ij}R+(1-\theta_{ij})A=A+\theta_{ij}(R-A)$. Using the relationship (11), R−A≈D/2, where *D* is the average branch divergence between two distinct founder samples, and furthermore that we can approximate D/2 as *T*, where *T* is the mean TMRCA between distinct founders. The branch relatedness we are working with is centered (7), so the constant term *A* cancels and we are left with $B_{i,j}\approx E[(\theta_{ij}-\theta_{iU}-\theta_{\;jV}+\theta_{UV})]T$. When samples are not closely related, $\theta_{iU}$, $\theta_{\;jV}$, and $\theta_{UV}$ are small, giving:


(13) $$B_{i,j}\approx \theta_{i,j}T,$$

where *T* is again the mean TMRCA for two random samples from the population (computable as one half of branch genetic divergence in tskit). The accuracy of this approximation depends strongly on too many factors to examine in depth here, including the length of the genome (a longer genome makes the average over inheritance in pedigree relatedness closer to the realized values) and the structure of the pedigree. In particular, this should work well if all pedigree founders are roughly equally related, and should best apply to individuals who are very closely related (e.g. first- and second-degree relatives). We examine this in practice below.

### Demonstration with the French–Canadian pedigree

**Fig. 1.**
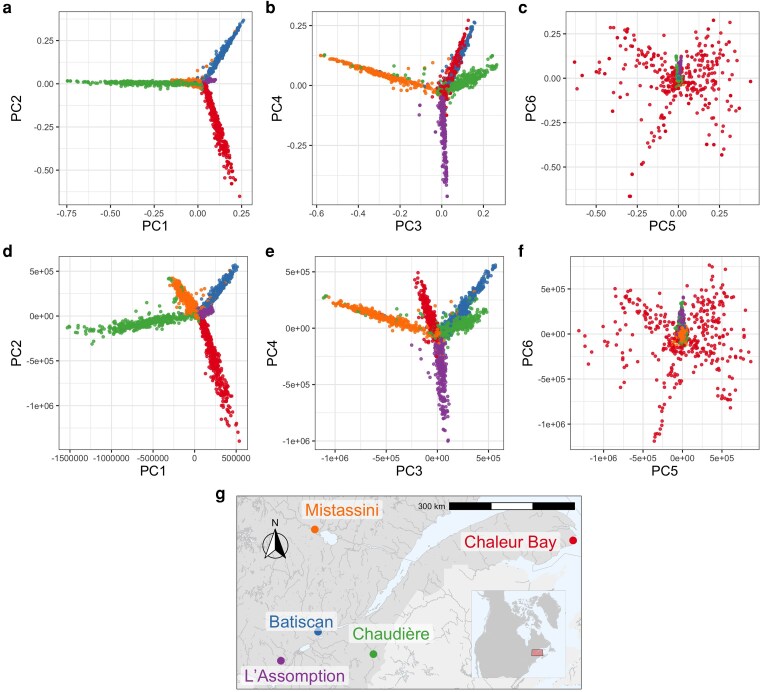
Principal components analysis (PCA) of pedigree and branch GRM of 2,321 French–Canadian individuals drawn from five different regions in Quebec. a)–c) The first six PCs of pedigree GRM. d)–f) The first six PCs of branch GRM. g) A partial map of Quebec with approximate locations of the five regions from which individuals were sampled. Both pedigree and branch PCA indicate highly similar population structure, concordant with the geographical separation of the five regions.

To empirically illustrate the connection between pedigree and branch GRM, we analyzed the pedigree of a subset of 2,321 individuals from the BALSAC dataset (Vézina & Bournival, 2020), drawn from five different regions in Quebec (Fig. 1g). For this subset, we computed pedigree and branch GRM. For the latter, we obtained an ARG from pedigree- and ancestry-informed simulation and computed the *whole-genome* branch GRM from the ARG. To evaluate the variance in branch GRM within a fixed pedigree, we repeated the simulations for a single chromosome, generating 100 ARGs for chromosome 3 only. See Methods for more details on the dataset and simulations performed.

The overall population structure according to pedigree and branch PCA of the respective centered GRMs of the 2,321 individuals is shown in Fig. 1, each showing sharp clustering by the five regions. Although all regions share a common bottleneck, over the last four centuries there has been a sufficiently little movement that each region pulls a distinct direction in PC space. In the pedigree PCA (Fig. 1a–c), roughly speaking, PCs 1 and 2 distinguish Chaudière, Chaleur Bay, and Batiscan; PCs 3 and 4 distinguish Mistassini, Chaudière, L’Assomption, and the combination of Batiscan and Chaleur Bay; and PCs 5 and 6 display variation within Chaleur Bay. The branch PCs also show the population structure (Fig. 1d–f), highly similar to that of the pedigree PCA. Some differences are evident, but no interpretation of this difference is obvious (to us). Note that PCs derived from the actual genomes, or an ARG estimated from the actual genomes, might well differ more strongly, if there were differences in the origins of settlers to the various regions – pre-pedigree structure for Fig. 1 was generated by simulation from an unstructured population.

**Fig. 2.**
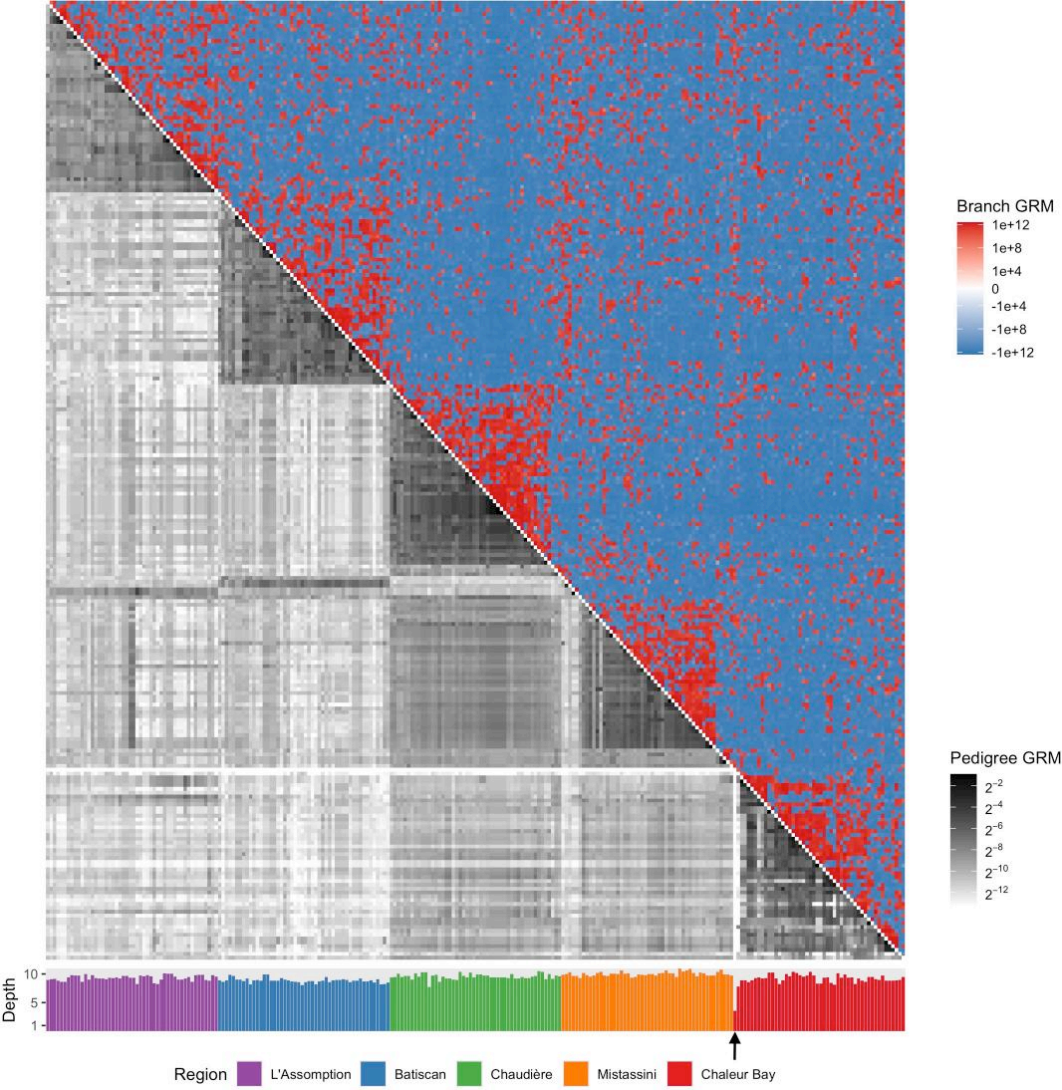
Comparison of pedigree relatedness and branch relatedness between 250 French–Canadian individuals from 5 regions in Quebec. Upper triangle: heatmap of the branch GRM computed from the ARG. Lower triangle: heatmap of the pedigree GRM. Bottom barplot: average founder depth in the pedigree for each individual. Both GRMs show broadly similar patterns, with higher relatedness values for pairs of individuals from the same region compared to those from different regions. The barplot indicates that all individuals have similar average founder depth, with the exception of one individual from Chaleur Bay (marked by the black arrow), who has near-zero values across the corresponding row and column of the pedigree GRM. Notes: The ordering of individuals is based on region and within region hierarchically on pedigree GRM. The branch GRM heatmap uses a “pseudo-log” scaling, which is a log scaling that transitions smoothly to a linear scale around zero. Because of the log scaling in the heatmap of the pedigree GRM, we added $10^{-4}$ to each entry to avoid issues with values close to zero.

**Fig. F1.**
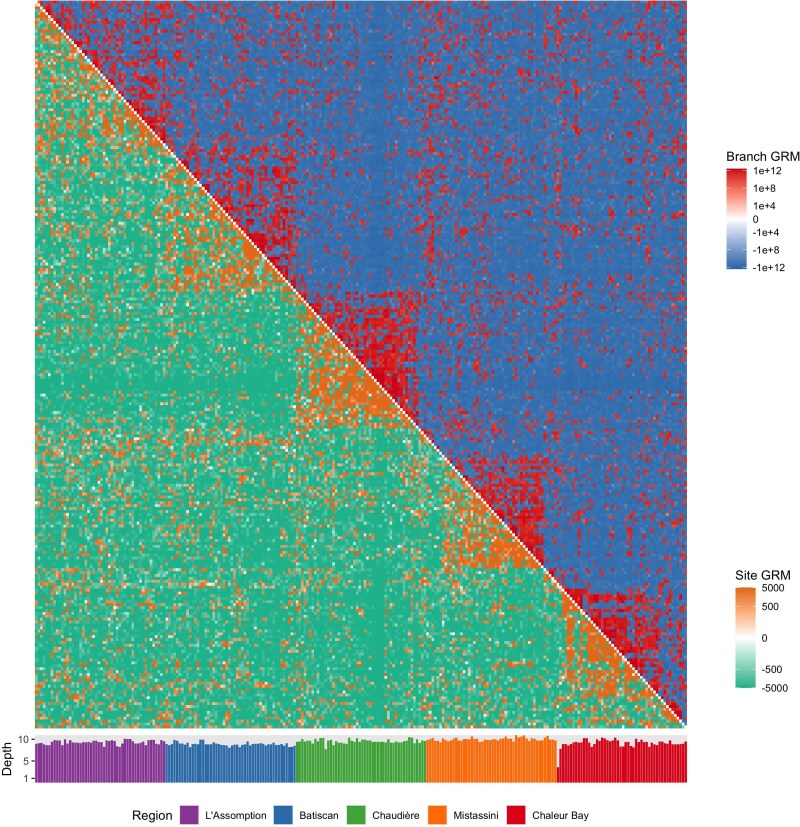
Comparison of genotype relatedness and branch relatedness between 250 French–Canadian individuals from 5 regions in Quebec. Upper triangle: heatmap of the branch GRM computed from the ARG. Lower triangle: heatmap of the genotype GRM. Bottom barplot: average founder depth in the pedigree for each individual. As with the pedigree GRM, both GRMs show broadly similar patterns, with higher relatedness values for pairs of individuals from the same region compared to those from different regions. Notes: The ordering of individuals is the same as that in Fig. 2 of the main manuscript. Both heatmaps uses a ’pseudo-log’ scaling, which is a log-scaling that transitions smoothly to a linear scale around zero.

Figure 2 compares the structure of the two GRMs themselves: branch above the diagonal, pedigree below. Again, they have similar but not identical structure. Notably, when individuals have a shallow pedigree (for example, one sampled individual from Chaleur Bay with an average founder depth of 3.5, indicated by an arrow in Fig. 2), their corresponding pedigree GRM values are low, which is not necessarily the case with branch relatedness (Anderson-Trocmé et al. 2023). Figure F1 in section Comparison of branch GRM and genotype GRM of the Appendix shows a similar heatmap comparing the genotype GRM with the branch GRM computed from the same ARG. The two GRMs are highly similar, as expected, since as discussed above, the branch GRM can be interpreted as the expected value of the genotype GRM under infinite-sites mutations.

**Fig. 3.**
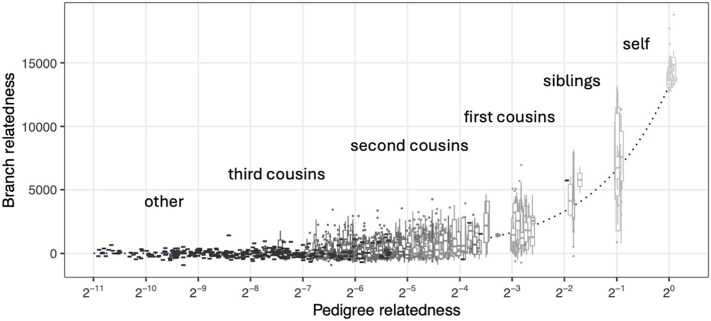
Variability in branch relatedness with respect to a fixed pedigree. Each box plot corresponds to a pair of individuals with pedigree kinship according to some types of (pedigree) relationships (self, siblings, etc.). The box plot for each pair of individuals depicts variation in branch relatedness across 100 simulated ARGs for chromosome 3 within the fixed pedigree. The dotted line indicates the approximate expected branch relatedness, which is the pedigree kinship multiplied by the mean TMRCA among pedigree founders.

**Fig. E1.**
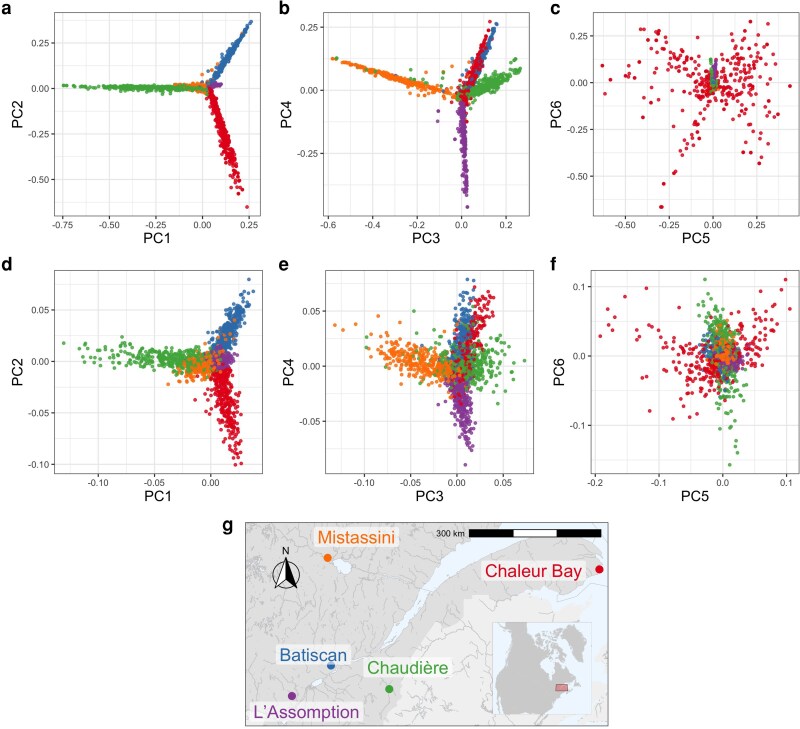
Principal components analysis (PCA) for branch (A-C) and pedigree (D-F) GRMs; all details are the same as in Fig. 1 except the results are for a single chromosome.

One reason that the branch PCA and the branch GRM plots are so concordant with the corresponding pedigree PCA and pedigree GRM plots (Figs. 1 and 2, respectively) is that they represent simulations of entire genomes, and so average over transmission of many chromosomes within the pedigree. Figure E1 shows that PCA produced from a single-chromosome simulation reflects geographic structure much less well than the whole-genome version in Fig. 1. This difference arises because random genetic inheritance, mutation, and ancestral variation produces differences between the pedigree and branch GRMs that are averaged out more effectively across more chromosomes (Weir et al. 2006; Hill and Weir 2011; García-Cortés et al. 2013; Thompson 2013). To further explore this variability—in other words, how the branch relatedness of a given pair of individuals varies across different instantiations of the genetic process—Fig. 3 shows branch relatedness values for a large number of pairs from 100 ARGs simulated for chromosome 3 within the pedigree. Each boxplot shows the range of values obtained between a *fixed* pair of individuals, across different simulations. As expected from equation (13), branch relatedness is approximately predicted by pedigree relatedness: boxplot midpoints lie on the dotted line, which shows the relationship (13) with *T* computed from the demographic model used for recapitation of the pedigree. The variation about this line is substantial, reflecting the wide range of realized genetic relatednesses between relatives on a single chromosome. The variability expected across a larger number of chromsomes would be smaller, scaling approximately with the square root of the number of chromosomes.

### Computation

We next present efficient algorithms for various operations, which are implemented in the tskit library (Ralph et al. 2020; Kelleher et al. 2024).

#### Computation of the entire branch GRM

As shown in equation (12), the branch GRM B is a straightforward function of the divergence matrix D, which describes the total branch length separating all pairs of samples. Because the output is a dense $n_{I}\times n_{I}$ matrix and at a minimum we must create and fill in the entries of this matrix, the complexity of this operation is at least $O(n_{I}^{2})$.

So-called “incremental algorithms”, which use the fact that the small changes in tree structure we observe due to recombination events often correspond to small changes in some accumulated statistic as we move along the genome, have led to very efficient algorithms in several contexts (Kelleher et al. 2016; Kelleher and Lohse 2020; Ralph et al. 2020; Grundler et al. 2025). The divergence matrix, however, does not easily lend itself to this approach. Incremental algorithms work well when we only need to consider the effects of inserting and removing edges on nodes that are *ancestral to* a given node. To compute the divergence matrix, however, we need to keep track of when the MRCAs of each pair of samples change, and this requires traversing the subtrees *descending from* nodes affected by edges being inserted and removed. Removing (or inserting) an edge changes the MRCA of all pairs between the set of samples descending from it and those not descending from it. In worst case (removing an edge to the root of a balanced binary tree with $n_{I}$ samples) this involves $O(n_{I}^{2})$ work per tree transition, and therefore the complexity of the operation is $O(n_{T}n_{I}^{2})$, where $n_{T}$ is the number of trees along the genome.

**Fig. G1.**
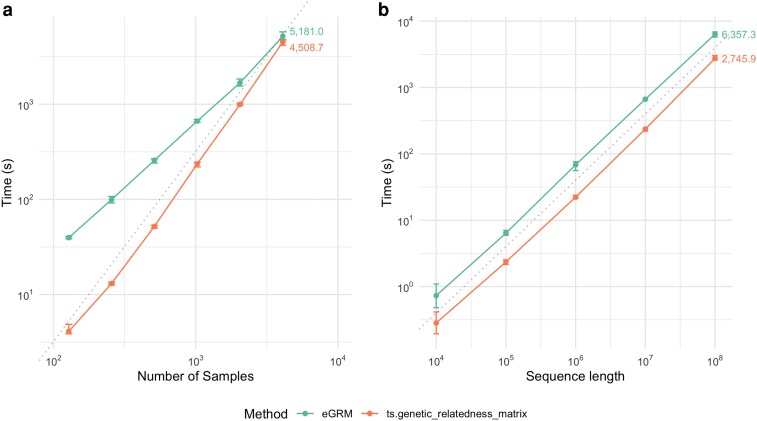
Time efficiency of different implementations of branch GRM computation (eGRM and ts.genetic_relatedness_matrix). Each dot corresponds to the average time taken across ten simulations with different random seeds. Error bars represent the range in time taken across the ten simulations. a) Branch GRM computation with genome sequence length fixed at $10^{7}$ and varying the number of samples. The grey dotted line indicates quadratic growth in time with respect to the number of samples. b) Branch GRM computation with number of sample nodes fixed at $2^{10}$ and varying genome sequence length. The grey dotted line indicates linear growth in time with respect to sequence length.

The naïve approach to this problem is to proceed tree-by-tree along the sequence, iterate over all (nI2) pairs of samples, compute the time to their MRCA, and update the corresponding element of D. MRCAs can be computed efficiently using the Schieber-Vishkin algorithm (Schieber and Vishkin 1988; Knuth 2011, pg. 164–167) which provides the MRCA of two nodes in constant time after an $O(n_{I})$ preprocessing step. The overall complexity is therefore $O(n_{T}n_{I}^{2})$, as we need to perform $O(n_{I}^{2})$ work for all $n_{T}$ trees. While this is the same complexity as the incremental approach outlined above, this “naïve” approach is in practice much faster than our attempts at an iterative algorithm, and is therefore the implementation used in tskit via the ts.genetic_relatedness_matrix method. The eGRM package (Fan et al. 2022) essentially uses the same approach, although implemented in Python and without efficient bulk MRCA queries. Their approach therefore requires $O(n_{T}n_{I}^{2}\log^{2}\;n_{I})$ time, as each MRCA query requires *O*(tree height) time, which is $\log\;n_{I}$ if the trees are balanced (Kelleher et al. 2016). Figure G1 in section Benchmarking branch GRM computations of the Appendix shows that the tskit implementation is faster than the eGRM implementation (Fan et al. 2022), although converging for larger sample sizes.

The $O(n_{T}n_{I}^{2})$ complexity of computing the branch GRM has significant implications for its utility in large-scale studies: quadratic algorithms are simply not feasible when we have millions of samples. The approximate mutation-dropping approach of Zhang et al. (2023) is not directly comparable to Fan et al. (2022) and our work. However, their follow-up work with the randomized Haseman-Elston method (Zhu et al. 2024) indicates that there are scalable computational approaches that can work with approximate branch GRMs. In the next section, we show instead how to perform matrix-vector operations with the branch GRM without materializing the actual matrix.

#### Computing branch GRM-vector products

In a wide range of applications, from science to engineering, many matrices are prohibitive to load explicitly. This has led to the development of so-called “matrix-free” algorithms that perform linear algebraic operations by accessing the matrix implicitly through matrix-vector products (Golub and Van Loan 2013). These include the conjugate gradient algorithm for solving linear systems, Krylov space methods for a wide range of linear algebraic operations which include the conjugate gradient algorithm and various matrix decompositions. A class of randomized algorithms have recently attracted attention and have demonstrated good performance in large scale problems (Murray et al. 2023). Here, we provide an algorithm that uses the tree sequence to efficiently multiply the genetic relatedness matrix by a vector, which allows us to use these powerful “matrix-free” algorithms. Many calculations with GRMs involve matrix-vector products (Colleau 2002; Colleau et al. 2017), ranging from PCA to estimating variance components (Wu and Sankararaman 2018; Lee et al. 2025). As an example, we will also describe how to efficiently perform principal component analysis (PCA) on the branch GRM (Halko et al. 2011).

Roughly, the algorithm is very efficient because although the GRM is not itself sparse or low rank, the ARG provides a decomposition of the GRM into a sum of low-rank components with hierarchical structure – the (sub)trees.

The biological intuition behind the algorithm leverages the shared haplotype structure implied by the ARG. In more detail, each edge represents a haplotype that is inherited by potentially many samples, and so it turns out that we can do the computation required by accumulating a “value” for each haplotype for as long as possible, only passing this value down to descendant nodes when the subtree below the edge changes. We provide the algorithm because it conveys intuition about this “shared haplotypes”, and points the way to other efficient algorithms. On the other hand, readers who are content with this high-level description can skip to the next section without missing important parts of the narrative.

To describe the algorithm, we first require some notation. Suppose now that $B_{i,j}$ is the uncentered branch relatedness between sampled genomes *i* and *j* as computed from the trees, that is, the sum of the areas of all branches in all trees that are ancestral to both *i* and *j*. This is $B_{i,j}=C\text{ov}[Z(i),Z(\;j)]$ – for simplicity, in this section we differ from the definition of equation (6) by omitting the centering terms. For a given vector w, we’d like to compute the matrix-vector product Bw. Write $b_{k}$ (k=0,…,K) for the unique recombination breakpoints on the genome including the start and end of the genome (the genome is a closed interval $\left[b_{0},b_{K}\right]$). Suppose that the $k^{\mathrm{th}}$ tree $T_{k}$ extends over the region from $b_{k}$ to $b_{k-1}$ along the genome, that the length of the branch (in units of time) above node *n* in $T_{k}$ is $\ell_{T_{k}}(n)=t_{\;p_{T_{k}}(n)}-t_{n}$, where $p_{T_{k}}(n)$ is the parent node of *n* in $T_{k}$. Finally, write $\leq_{T}$ for the partial ordering of nodes induced by inheritance in tree *T* with older nodes larger than younger nodes. Then, the uncentered branch relatedness matrix is


(14) $$B_{i,j}=\sum_{k}(b_{k}-b_{k-1})\sum_{n\;:\;i,j\leq_{T_{k}}n}\ell_{T_{k}}(n).$$

The $i^{\mathrm{th}}$ element of the matrix-vector product Bw is therefore


(15) $$\begin{array}{rl} (Bw{)}_{i} & =\sum_{j}B_{i,j}w_{j}\\ & =\sum_{j}\left(\sum_{k}(b_{k}-b_{k-1})\sum_{n\;:\;i,j\leq_{T_{k}}n}\ell_{k}(n)\right)w_{j}\\ & =\sum_{k}(b_{k}-b_{k-1})\sum_{n\;:\;i\leq_{T_{k}}n}\ell_{T_{k}}(n)\sum_{\;j\;:\;j\leq_{T_{k}}n}w_{j}\\ & =\sum_{k}(b_{k}-b_{k-1})\sum_{n\;:\;i\leq_{T_{k}}n}\ell_{T_{k}}(n)w_{k}(n) \end{array}$$

Here $\left\{n\;:\;i,j\leq_{T_{k}}n\right\}$ is the set of nodes *n* that are ancestral to both *i* and *j* in $T_{k}$, and $\left\{n\;:\;i\leq_{T_{k}}n\right\}$ are those nodes ancestral to *i*. The new variable $w_{k}(n)$ is the sum of sample weights below *n* in tree $T_{k}$:


(16) $$w_{k}(n)=\sum_{u\;:\;u\leq_{T_{k}}n}w_{u},$$

which is a familiar term from Ralph et al. (2020). Although a single entry of Bw could be computed efficiently from the algorithm in Ralph et al. (2020), it does not scale well because it requires a separate set of weights for each entry of the vector.

We present an efficient algorithm for computing the entire matrix-vector product. The general idea is simple: as we move left-to-right along the tree sequence, we keep track of two quantities for each node *n*: the *weight*  w(n) of the node in the current tree ($w_{k}(n)$ above) and the *value*  v(n) of the haplotype carried by *n*, which will contribute to all descendants of *n*. Additionally, we keep track of the last *position*  x(n) in which the node was updated. As we move along the genome, we update any nodes ancestral to any changes in the tree: all other nodes are the roots of unchanged subtrees and hence remains unchanged. Each edge contributes to potentially many entries in the output vector, so by accumulating values of haplotypes, we reduce the amount of necessary work.

##### Algorithm V

(*Branch GRM-vector product*). Given values $w_{i}$ for $1\leq i\leq n_{I}$, a sequence of positions that are recombination breakpoints $b_{k}$ for 1≤k≤K along the genome and corresponding sequences of edges to remove ($R_{k}$) and add ($A_{k}$) at each position, compute the values $y_{i}=\sum_{j}\;B_{i,j}w_{j}$ for $1\leq i\leq n_{I}$, assuming all samples are leaves in all trees. Let *T* be the current tree, $\ell_{T}(n)=t_{\;p_{T}(n)}-t_{n}$ be the length of the edge above *n* in *T* (or zero, if *n* has no parent), initialize k=1, x(n)=0, and v(n)=0 for all n∈V. Set $w(i)=w_{i}$ for each sample *i*, and w(n)=0 for all other nodes. Let $z(n)=\ell_{T}(n)(b_{k}-x(n))$ be a function computed from the current values of *k* and *x* at all times.

**V1.** [Remove edges] For each edge $(c,p)\in R_{k}$, and for each node $n\geq_{T}p$, set v(n)+=z(n)w(n), then w(n)−=w(c), v(c)+=v(n), and $x(n)=b_{k}$. Then, set $x(c)=b_{k}$ and remove the edge from *T*.
**V2.** [Add edges] For each edges $(c,p)\in A_{k}$, and for each node $n\geq_{T}p$, set v(n)+=z(n)w(n), then w(n)+=w(c), v(c)−=v(n), and $x(n)=b_{k}$. Then, set $x(c)=b_{k}$ and add the edge to *T*.
**V3.** [Iteration] If k<N, set k+=1 and return to V1. Otherwise, set $y_{i}=v(i)$ for $1\leq i\leq n_{I}$ and finish.

Algorithm **V** follows a similar structure to previous incremental algorithms (Kelleher et al. 2016; Ralph et al. 2020): at each tree transition we update some global state to account for the insertion and removal of the edges affected. Here, the overall goal is different: rather than keeping track of some cumulative value among the nodes in a given subtree (say, total branch length) we are instead keeping track of the total contribution to each node from nodes *ancestral to it*. By some subtle bookkeeping, we can keep track of the cumulative contribution to each node, in only updating each node when it is affected by an edge insertion or removal. Each node accumulates the contributions that are passed down from above until an edge below it is added or removed. At each edge insertion or removal v(n) is updated by traversing up to the root of the current subtree (also keeping the weights w(n) up to date), and the accumulated contribution passed down to the child node of the edge *c*. Finally, we set x(c) to $b_{k}$ (the current position) to mark the last position this node was updated.

The above explanation is a rough sketch of the algorithm. A full proof of correctness is provided in section Proof of correctness of Algorithm V of the Appendix. The algorithm has been implemented in the ts.genetic_relatedness_vector method in tskit, somewhat generalized to include centering and to allow for samples that are not leaves, and is extensively tested.

**Fig. I1.**
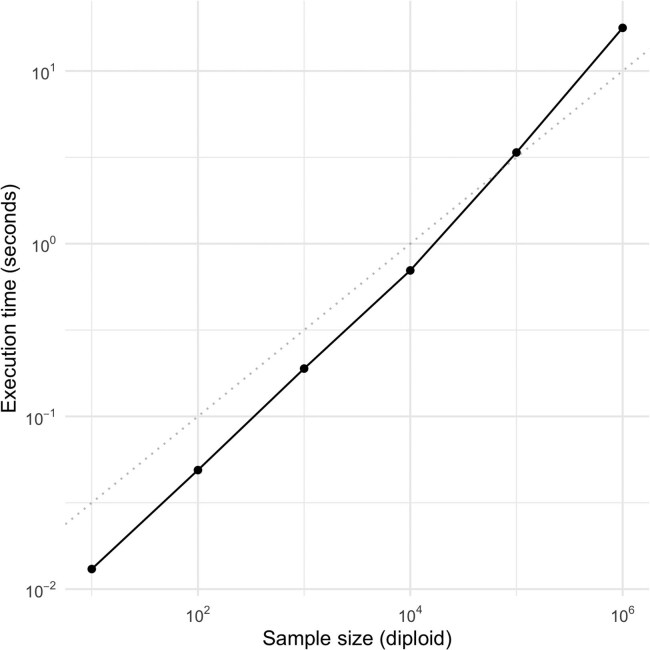
Computational scaling of the branch GRM-vector product algorithm implemented in tskit for subsets of a large simulation of French–Canadians. For reference, the grey dotted line indicates square root growth in time with respect to sample size (an arbitrary choice).

The analysis of this algorithm is straightforward and follows a standard pattern (Kelleher et al. 2016; Ralph et al. 2020). Because recombination results in a small modification of the current tree, each tree transition incurs O(1) edge removals and insertions. Each edge removal in step **V1** involves examining only nodes ancestral to the edge, and therefore incurs a cost of $O(\log\;n_{I})$, assuming trees are balanced. Edge insertions in **V2** have the same cost. Thus, as the first tree requires inserting $O(n_{I})$ edges requiring O(1) work, the overall complexity is $O(n_{I}+n_{T}\log\;n_{I})$. This logarithmic time complexity is borne out in Fig. I1 of section Computational scaling of the branch GRM-vector product algorithm where we plot the time taken to compute the branch GRM-vector product against subsets of a large simulated ARG (Anderson-Trocmé et al. 2023) of chromosome 21. Here, it takes only 17.8 s to run the ts.genetic_relatedness_vector method on the ARG with 1 million diploid samples (6,694,080 nodes; 31,840,754 edges; 4,013,273 trees). In contrast, computing the full branch GRM using the ts.genetic_relatedness_matrix method for the ARG with ten diploid samples (61,412 nodes; 297,171 edges; 93,543 trees) required 28 seconds.

#### Branch PCA

The principal components (PCs) of the branch GRM can be obtained using randomized SVD (Halko et al. 2011 ), a method that can find the eigenvectors of a matrix that is only implicitly defined through a matrix-vector multiplication. We implemented the algorithm as ts.pca in tskit.

##### Algorithm rPCA

(*Randomized PCA of branch GRM*). Let B be the branch GRM for $n_{I}$ individuals, let *k* be the desired number of PCs, and *q* the number of iterations. Multiplying B with a vector is done by Algorithm **V**.

**P1.** [Range estimation] Sample a random matrix $\Omega \in R^{n_{I}\times k}$ in which the entries are independent standard normal variables. Obtain a basis matrix $\;Q\in R^{n_{I}\times k}$ by applying QR decomposition to BΩ. Update the basis matrix *q* times, setting Q to the result of a QR decomposition for BQ, where Q is from the previous iteration.
**P2.** [Small singular value decomposition] Compute $\;W=\;Q^{⊺}\;B$ and obtain the singular vectors $\;U\in R^{k\times k}$ by exact singular value decomposition of W. Then the columns of $\;Q\;U\in R^{n\times k}$ contain the desired PCs of the branch GRM B.

The algorithm has two advantages over directly applying the exact SVD to the branch GRM. It needs less time and memory because the $n_{I}\times n_{I}$ branch GRM is never computed or stored. The algorithm extracts the relevant information through the efficient matrix-vector product Algorithm **V**. Secondly, the exact SVD is applied to an $n_{I}\times k$ matrix, where *k* is much smaller than $n_{I}$. This reduces the amount of computation considerably.

**Fig. 4.**
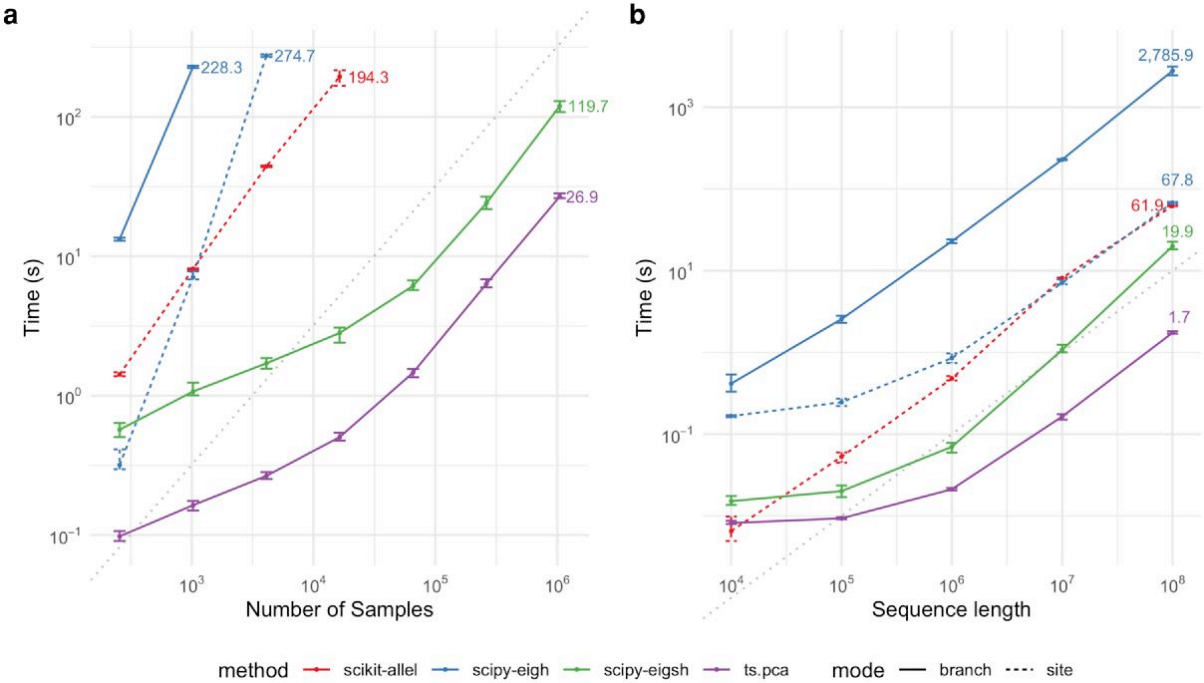
Time efficiency of different implementations of PCA. Each dot corresponds to the average time taken across ten simulations with different random seeds, and error bars represent the range in time taken across the ten simulations. Methods are: scikit-allel’s built-in PCA method using the genotype matrix; scipy’s eigenvector-finding method eigh applied to the full GRM; scipy’s implicit eigenvector-finding method eigsh given the genetic_relatedness_vector function; and ts.pca, the implementation of Algorithm **V** in tskit. a) PCA with genome sequence length fixed at $10^{7}$ and varying the number of samples. b) PCA with number of sample nodes fixed at $2^{10}$ and varying genome sequence length. Branch mode refers to branch PCA and site mode refers to genotype PCA. For reference, in both figures the grey dotted lines indicate linear growth (with slope 1 and an arbitrary intercept).

The efficiency of the branch PCA algorithm and the underlying branch GRM-vector product algorithm are illustrated in Fig. 4. See Methods for details of the benchmarking methodology. We observed significant benefits from using implementations that avoided the storage of the GRM or genotype matrix in memory, particularly for larger numbers of samples (Fig. 4). Notably, ts.genetic_relatedness_matrix failed due to memory limits when computing the branch GRM for $2^{12}=4096$ sample nodes and when computing the genotype (site) GRM for $2^{14}=16384$ sample nodes. Randomized PCA on the genotype matrix in scikit-allel failed due to memory limits for $2^{16}=65536$ sample nodes. Implementations that relied solely on the implicit matrix-vector product using tskit were substantially more efficient: both ts.pca and eigsh from scipy using ts.genetic_relatedness_vector as a linear operator were able to scale to $2^{20}=1,048,576$ samples. The native implementation of ts.pca consistently outperformed eigsh, with the relative difference decreasing slightly with the number of samples, and increasing with sequence length. Our method achieved compute times of seconds for even large samples on large genomes: for example, on a genome of $10^{7}$bp, ts.pca took on average 0.27s for $2^{12}=4,096$ samples and 26.9s for $2^{20}=1,048,576$ samples, while eigsh took 1.7s for $2^{12}=4,096$ samples and 119.7s for $2^{20}=1,048,576$ samples. This difference primarily reflects the differences in the underlying algorithms used for PCA: ts.pca uses a randomized SVD while eigsh uses the implicitly restarted Lanczos method (Lehoucq et al. 1998).

## Methods

### French–Canadian pedigree

To demonstrate the similarities and differences between pedigree and branch relatedness, we performed a range of analyses on a subset of an extended pedigree of French–Canadian individuals from the BALSAC project (Vézina and Bournival 2020). Spanning over 400 years, this pedigree is compiled from over 4.5 million parish records across Quebec. In this paper, we restricted our analyses to five regions, each containing five neighboring parishes (Table 2). Using the migratory patterns from Anderson-Trocmé et al. (2023) as a reference, we identified five distinct regions and sampled individuals from parishes within each region to minimize excessive relatedness within each sampling unit while also mitigating the risk of de-anonymization. Our data access agreements with BALSAC dictated that we use parish records of more than one hundred years old (before 1924) to publish their metadata and summary statistics. As a result, the pedigree used in this study contains ascending genealogy for 500 randomly chosen contemporary individuals from each of the five regions, with individuals sampled across the five selected parishes per region.

**Table 2.** Selected regions and parishes from the BALSAC French–Canadian Pedigree.

Region	Parishes	Approximate location
Chaleur Bay	St Michel, St François De Sales, St Georges De Malbaie, St Pierre De Malbaie, and St Joseph	48.5222, −64.2156
Batiscan	Ste Geneviève De Batiscan, St Luc De Vincennes, St Narcisse, and St Stanislas	46.5324, −72.3398
Chaudière	St Georges, St Benoit Labre, St Philibert, St Come, and St Martin De Tours	46.1184, −70.6691
L’Assomption	St Jacques, St Alexis, Ste Marie Salomée, and St Esprit	45.9483, −73.5702
Mistassini	St Félicien, St Méthode, Notre Dame De La Dore, St Cyrille, and Ste Lucie	48.6399, −72.4543

The sub-pedigree obtained from the selected five regions, each containing five parishes, consisted of 61,490 individuals, including 2,321 probands. A subset of the probands exhibited a low depth of pedigree, reflecting an incomplete pedigree. To ensure meaningful comparisons, we computed the maximum pedigree depth for each individual and derived an average depth metric. A total of 48 probands with an average depth of less than 3 were excluded due to their shallow pedigree. After this filtering step, a total of 2,273 probands were retained for downstream analyses.

We computed pedigree and branch relatedness between individuals of interest. Pedigree relatedness was computed after Lange and Sinsheimer (1992) and Colleau (2002). Branch relatedness was calculated from an ARG obtained with the simulation based on pedigree and ancestry described in Anderson-Trocmé et al. (2023). In short, this simulation uses msprime (Baumdicker et al. 2022) for a backward in time simulation in two stages. First, it samples chromosomal inheritance through the fixed pedigree to obtain an ARG within the pedigree. Second, it simulates the ancestry of the ARG obtained in the first stage by coalescent simulation from a given demographic model (i.e. “recapitation”). We simulated an ARG for each of the 22 autosomes and computed the whole-genome branch GRM by summing the branch GRMs across each chromosome. To study the stochastic variation in recombination and coalescence events within the pedigree, we simulated 100 replicates of an ARG for chromsome 3 using different random seeds. We chose to simulate only the complete human chromosome 3 due to its large size, while reducing the overall computational cost. This approach allowed us to assess the variance in branch GRM while maintaining consistency with the underlying pedigree.

To explore the overall population structure within the pedigree and the simulated tree sequences, we performed PCA on the set of 2,273 probands. For pedigree PCA, we first computed the pedigree GRM among the probands and then eigen-decomposed the centered GRM using eigh function from scipy (Virtanen et al. 2020). For branch PCA, to avoid undue influence of large, low-recombination regions, we first remapped genomic coordinates from base pairs to genetic distance, using the HapMap II genetic map provided by stdpopsim (Adrion et al. 2020). We then used Algorithm **V** to define linear operators to compute whole GRM-vector products for each of the 22 autosomes. We then defined a whole-genome linear operator by taking the sum of each of the autosomal linear operators. This whole-genome linear operator was used with the eigsh function in scipy.sparse.linalg module to obtain the first six PC of the whole-genome GRM.

To compare pedigree and branch relatedness for specific pairwise relationships, we focused our attention on a single subset of the 2,273 probands. Specifically, we randomly sampled one parish per region and subsampled at least five siblings, first cousins, second cousins, and third cousins from each parish. We then subsampled additional individuals from each parish to obtain a total of 50 individuals per parish. With these 250 individuals, we computed the pedigree GRM and a branch GRM for each of the 100 simulated ARGs of chromosome 3.

### Benchmark simulations

We assess the computational efficiency of our implementations for branch GRM and PCA, with simulations, recording the time for calculations for a range of tree sequences. We simulated the tree sequences with msprime (Baumdicker et al. 2022), and varied either the genome sequence or the number of sample nodes (haploid individuals). All computations were carried out on a single CPU with 4GB of RAM.

#### Branch GRM

We compared ts.genetic_relatedness_matrix for computing the branch GRM to the implementation in the eGRM package (Fan et al. 2022). The default values for simulations parameters were $10^{7}$ for the genome sequence length, $2^{10}$ for the number of samples, and effective population size of $10^{4}$. We then varied genome sequence length from $\left(10^{4},10^{5},10^{6},10^{7},10^{8}\right)$ and the number of samples from $\left(2^{7},2^{8},2^{9},2^{10},2^{11},2^{12}\right)$, each one at a time. For each simulation setting, we generated 10 tree sequences with different random seeds and reported the average time taken to compute the GRM with each implementation.

#### Branch PCA

We assessed our branch PCA Algorithm rPCA against a number of comparators using scipy (Virtanen et al. 2020) and scikit-allel (Miles et al. 2024): (1) Calculating branch GRM using ts.genetic_relatedness_matrix followed by eigenanalysis using eigh function from scipy. (2) Eigenanalysis of branch GRM using eigsh function from scipy using the implicit matrix-vector product Algorithm **V** as a linear operator. (3) Calculating genotype GRM using equation (3) followed by eigenanalysis using eigh function from scipy. (4) Randomized PCA of genotype matrix using randomized_pca function from scikit-allel. We used the same simulations as for the branch GRM computation benchmark, but explored larger sample sizes, ranging across $\left(2^{8},2^{10},2^{12},2^{14},2^{16},2^{18},2^{20}\right)$. For each simulation setting, we generated 10 tree sequences with different random seeds and reported the average and range of time for PCA with each implementation.

## Discussion

Recent advances in ARG inference have generated significant interest in leveraging ARGs for genetic analyses. In this paper, we examined the relationship between different definitions of genetic relatedness in the common context of additive traits on an ARG, especially the emergent notion of “branch” relatedness. We also demonstrated how branch relatedness compares with pedigree relatedness in simulations through a pedigree of French–Canadians. We then described an algorithm to use the tree sequence to compute the product of the branch GRM with a vector, to bypass the fundamental problem of quadratic complexity of computing and storing GRMs. This algorithm allowed us to use randomized linear algebra methods for branch PCA using an ARG on a million samples in 30 seconds and less than 4GB of RAM.

The described branch relatedness unifies several notions of relatedness into one framework by leveraging the ARG encoding of how sampled genomes relate to each other. One thing that distinguishes these notions of relatedness is which aspects of genetics and genealogy are unobserved or observed, and which are averaged over or fixed. For instance, pedigree relatedness averages over recombinations within a known pedigree, while genotype relatedness averages over typed loci stored in a genotype matrix. Our branch relatedness is conceptually equivalent to the eGRM from Fan et al. (2022) and the ARG-GRM from Zhang et al. (2023), although we omit the scaling by $(p(1-p){)}^{\alpha }$ used by both. The “e” in eGRM denotes expectation of the genotype GRM under a Poisson mutation process along ARG branches. At the risk of further confusing terminology, we adopted the term *branch* to highlight that this measure of similarity is derived from the extent of shared branch area between individuals, explicitly distinguishing it from expectations that are conditional on other quantities – for instance, expected covariance given a pedigree or expected covariance given a collection of genotypes (but not their effect sizes). We have seen that branch relatedness varies substantially for relatives of a given degree, in line with the theory on pedigree and genetic ancestors (Chang 1999; Weir et al. 2006; Hill and Weir 2011; García-Cortés et al. 2013; Thompson 2013).

Does mutation contribute a large degree of variability in addition to the branch relatedness? Fan et al. (2022) computed the “varGRM” to describe this (for general theory see Ralph 2019); and in general the answer is “no”—randomness due to the mutation process adds little variability beyond recombination, except to small segments of the genome.

In our analysis of the BALSAC dataset, we simulated ARGs from a fixed pedigree to investigate the relationship between pedigree relatedness and branch relatedness. In practice, however, the true ARG is not directly observable and must instead be inferred from sequence data, which can introduce additional sources of variability. A growing number of ARG inference methods are now available, varying in their computational scalability and overall accuracy (see Nielsen et al. 2024 for a recent review). Importantly, the reliability of downstream statistics derived from the ARG may depend less on global reconstruction accuracy and more on the accurate estimation of specific features (Peng et al. 2025). For branch relatedness in particular, correctly estimating branch lengths, i.e. coalescence times, deep in the past is especially crucial, as many pairs of individuals share common ancestry at these more ancient branches. Fan et al. (2022) conducted a simulation study comparing two ARG inference methods - Relate (Speidel et al. 2019) and tsinfer+tsdate (Kelleher et al. 2019; Wohns et al. 2022) - for their ability to estimate the branch GRM (their eGRM). They found that Relate performs better in this context, albeit at greater computational cost; subsequent updates to the algorithms may change this.

We have chosen to interpret the GRM in the context of a generative model of traits following the initial definition of relatedness (Wright 1922). However, the worth of a given GRM is determined by how well it works in practice, rather than its theoretical justification, and applications have motivated a number of interpretations and adjustments (Speed and Balding 2015). However, adjusting the trait model gives a natural setting in which to suggest extensions and the corresponding GRM is then a by-product of these extensions. Although our current definition of relatedness assumes equal prior effects across all loci, one could consider alternatives whereby we incorporate prior information on effect sizes. For example, selection reduces frequency of deleterious mutations with strong effects from the population and such mutations may justify a different prior; this prior might depend on mutation age in a similar way that the GRMs often weight alleles by a function of their frequency (Speed and Balding 2015). Functional annotations have been used to improve fine-mapping and genomic prediction (e.g. MacLeod et al. 2016; Weissbrod et al. 2020, 2022) and could be incorporated as prior information on mutation effects, which will refine branch relatedness calculations for trait-based analyses.

Computing a full GRM is inherently a quadratic operation and therefore not feasible on large sample sizes. It is possible, however, to calculate GRM-vector products at a substantially lower computational cost. With $n_{I}$ samples and $n_{T}$ local trees, our branch GRM-vector product algorithm has complexity $O(n_{I}+n_{T}\log\;n_{I})$. This relies on two insights: first, we use local trees to efficiently encode the low-dimensional block structure of the contribution to the GRM of a single local tree; and second, we leverage the fact that most tree structure is shared across many local trees in the ARG. This removes the need for approximate methods such as the Monte Carlo sampling of mutations on the ARG used by Zhang et al. (2023). The method is probably most similar to Zhu et al. (2024, Algorithm S4), who use iterative algorithms to compute GRM-vector multiplication with the genotype GRM from Monte Carlo-sampled mutations. Since Zhu et al. (2024) do not provide an asymptotic analysis of their algorithm, it is not straightforward to compare our method with theirs. Their implementation assumes that the subtree below each node does not change, thus allowing a single top-down traversal. This requirement of unique nodes per subtree (also made by Nowbandegani et al. 2023 and DeHaas et al. 2024) is convenient for graph traversal algorithms, but in our experience can lead to substantially larger file sizes. We also provide a highly efficient vector-GRM-vector product algorithm, similar to the classic algorithm for large pedigrees (Colleau 2002), using the generic framework of Ralph et al. (2020).

This work provides the definition of branch relatedness based on a concrete trait model, algorithms to efficiently compute with the corresponding branch GRM for millions of genomes, and well documented and thoroughly tested open-source tskit implementation. These contributions point the way towards future mega-scale population genetics and quantitative genetics. The clear definition of branch relatedness (based on the fundamental ARG encoding of sampled genomes, a trait model extendable with additional biological prior information) could enhance the analyses of diverse and admixed genomic datasets that are challenged by many evolutionary processes and data availability (e.g. MacLeod et al. 2014 ; Martin et al. 2017; Durvasula and Lohmueller 2021; Ros-Freixedes et al. 2022a; Wang et al. 2022; Yair and Coop 2022). The efficient branch GRM-vector product algorithm will speed up analyses of population structure, genome-wide associations, heritability, and genomic prediction.

## Data Availability

We thank the BALSAC project for providing access to their genealogical data and for their guidance in selecting an appropriate subset of the genealogy for our analyses. The data that support the findings of this study are available upon request from the BALSAC Project. Access to the data is restricted by ethical regulations surrounding the use of population data for scientific research. Contact BALSAC for more information and to apply for access to these data (https://balsac.uqac.ca/).

## Code availability

All code used to generate the results and figures in this paper is available at https://github.com/brieuclehmann/tsrelatedness.

## Appendix A: Treatment of reference alleles

In the model above we have set the effect of a given allele (either the reference, major, or ancestral allele) to zero, and assigned the effect of each other allele an independent Gaussian effect. This, however, apparently depends on the choice of reference allele, and so another choice would have been to assign a separate independent Gaussian effect to *every* allele. It turns out that this is only equivalent in the biallelic case, in the sense that there exists a transformation of parameters for one model that produces the other model. The situation is essentially that of choosing the contrasts for a factor when fitting a linear model with Gaussian priors on the parameters: the BLUPs are the same, but the posterior distributions on the parameters may not be, because different choices of contrasts include non-equivalent priors. We have made the allele-symmetric choice because it is insensitive to the choice of reference allele and because it fits more naturally in existing schemes for computing with tree sequences.

To see this, consider the (contrived) case in which we have *n* haploid samples genotyped at a single locus, and every sample has a distinct genotype. If we write $Z_{k}$ as the effect of genotype *k*, and *Y* for an intercept, then $X_{k}$, the trait value of sample *k* is:

$$\begin{array}{rl} X_{k} & =Y+Z_{k}\\ & =Y+Z_{0}+(Z_{k}-Z_{0}). \end{array}$$

Now, the two models are:

(A1 )
$$\begin{array}{cc} Y & ∼\text{Normal}(0,\alpha )\\ Z_{k} & ∼\text{Normal}(0,\beta ), \end{array}$$

and

(A2 )
$$\begin{array}{cc} Y+Z_{0} & ∼\text{Normal}(0,\gamma )\\ Z_{k}-Z_{0} & ∼\text{Normal}(0,\delta ). \end{array}$$

Under model (A1), the covariance matrix of *X* is

$$C_{1}=\left[\begin{array}{cccc} \alpha +\beta & \alpha & \cdots & \alpha \\ \alpha & \alpha +\beta & \alpha & \vdots \\ \vdots & \alpha & \ddots & \alpha \\ \alpha & \cdots & \alpha & \alpha +\beta \end{array}\right].$$

On the other hand, under model (A2), the covariance matrix of *X* is

$$C_{2}=\left[\begin{array}{cccc} \gamma & \gamma & \cdots & \gamma \\ \gamma & \gamma +\delta & \gamma & \vdots \\ \vdots & \gamma & \ddots & \gamma \\ \gamma & \cdots & \gamma & \gamma +\delta \end{array}\right].$$

For n=2 and a given *α* and *β*, we can choose *γ* and *δ* so that the two matrices are the same; however, this is not in general possible for n>2, i.e. in the more-than-biallelic case.

One might hope that even though the two models are not equivalent for $\left(X_{i}\right)$, they can be made equivalent for the centered values $\left(X_{i}-\bar{X}\right)$. However, one can check that this is not the case: in model (A2), the variance of $X_{0}-\bar{X}$ (the ancestral-allele-carrying sample) differs from the other samples, while under model (A1) they are the same. To see this, compare $PC_{1}P$ and $PC_{2}P$, with $P=I-11^{T}/n$.

## Appendix B: Proof of equation (4)

As in the main text, *i* and *j* are fixed haploid individuals, while *U* and *V* are uniformly chosen haploid individuals (chosen with replacement, so it may be that i=U=V, for instance). Now, form the random variable $\left(X_{i},X_{j},X_{U},X_{V}\right)$ that takes the value $\left(G_{i,\ell },G_{\;j,\ell },G_{U,\ell },G_{V,\ell }\right)$ with probability $\frac{1}{\left(n_{I^{2}}n_{L}\right)}$ for $U,V=1,\ldots ,n_{I}$ and $\ell =1,\ldots ,n_{L}$. In other words, we choose the individuals *U* and *V* uniformly at random, with replacement, from the set of $n_{I}$ individuals, and also choose a locus ℓ uniformly at random from the set of $n_{L}$ loci; then $\left(X_{i},X_{j},X_{U},X_{V}\right)$ is the alleles of those individuals at that locus. In the following, we will repeatedly use the fact that $G_{i,\ell }^{2}=G_{i,\ell }$ (since $G_{i,\ell }\in \{0,1\}$). Conditional on locus ℓ, we have:

$$\begin{array}{rl} P(X_{i}=X_{j}\;|\;\ell ) & =(1-(G_{i,\ell }-G_{\;j,\ell }{)}^{2})\\ & =1-G_{i,\ell }-G_{\;j,\ell }+2G_{i,\ell }G_{\;j,\ell },\\ P(X_{i}=X_{U}\;|\;\ell ) & =\frac{1}{n_{I}}\sum_{u=1}^{n_{I}}(1-(G_{i,\ell }-G_{u,\ell }{)}^{2})\\ & =\frac{1}{n_{I}}\sum_{u=1}^{n_{I}}(1-G_{i,\ell }-G_{u,\ell }+2G_{i,\ell }G_{u,\ell })\\ & =1-G_{i,\ell }-p_{\ell }+2G_{i,\ell }p_{\ell },\\ P(X_{U}=X_{V}\;|\;\ell ) & =\frac{1}{{n_{I}}^{2}}\sum_{u=1}^{n_{I}}\sum_{v=1}^{n_{I}}(1-(G_{u,\ell }-G_{v,\ell }{)}^{2})\\ & =\frac{1}{{n_{I}}^{2}}\sum_{u=1}^{n_{I}}\sum_{v=1}^{n_{I}}(1-G_{u,\ell }-G_{v,\ell }+2G_{u,\ell }G_{v,\ell })\\ & =1-2p_{\ell }+2p_{\ell }^{2}. \end{array}$$

Combining these expressions, we have the following identity:

$$\begin{array}{rl} 2(G_{i,\ell }-p_{l})(G_{\;j,\ell }-p_{l}) & =P(X_{i}=X_{j}\;|\;\ell )-P(X_{i}=X_{U}\;|\;\ell )\\ & \;-P(X_{j}=X_{V}\;|\;\ell )+P(X_{U}=X_{V}\;|\;\ell ). \end{array}$$

Note the similarity to expression (2). Since ℓ is chosen uniformly at random, it follows that:

(B1 )
$$\begin{array}{rl} C_{i,j} & =\frac{1}{2}\left(P(X_{i}=X_{j})-P(X_{i}=X_{U}\right)\\ & \;-P(X_{j}=X_{V})+P(X_{U}=X_{V})). \end{array}$$

This is expression (4).

## Appendix C: Multi-allelic loci in haploids

Following from the methods in the main paper, here we expand the haploid case with two alleles to multiple alleles. The allele of individual *i* at locus *l* is $G_{i,l}\in A$ for some alphabet A, and each allele *a* at each locus *l* has an additive effect $Z_{l,a}$. We have this information for $n_{l}$ loci, $n_{a}$ distinct alleles, and $n_{i}$ haploid individuals. (Here we take the alphabet to be the same for all loci, but this is only for convenience, because alleles not present at a locus have no effect.) Recall that the genetic value of individual *i* is:

$$Z(i)=\frac{1}{p}\sum_{g=1}^{p}\sum_{\ell =1}^{n_{\ell }}Z_{\ell ,G_{i,\ell ,g}},$$

where each allele *a* at each locus ℓ has an independent effect $Z_{\ell ,a}$, with mean 0 and variance $\sigma^{2}$. However, this might seem not very well defined, since addition of invariant sites affects the result. So, suppose at each locus there is an ancestral allele whose effect is zero.

We can define the covariance for this case using equation (4). To write this covariance as a sum over alleles, it will be convenient to use the following lemma.

Lemma C1 Let a,b,c,d∈{0,1}, and let [a=b]=1 if a=b and [a=b]=0 otherwise. Then we have:

(C1 ) 2(a−c)(b−d)=[a=b]−[b=c]−[a=d]+[c=d].

Proof. First, notice that both sides are equal to 0 if a=b=c=d or if three agree and only one differs. On the right-hand side, this occurs because each of a,b,c,d appears in two terms, one positive and one negative. Therefore, if any three agree and differ from a fourth one, then we are left with 1−1−0+0=0. Now consider the case of two pairs. If a=b≠c=d, then both sides are equal to +2. If a=d≠b=c, then both sides are equal to −2. Finally, if a=c≠b=d, then both sides are 0.

Now, for each a∈A let $X_{i,l}^{a}=1$ if $G_{i,l}=a$, and equal 0 otherwise. We have the following identity between individuals *i* and *j*:

$$\sum_{a}[X_{i,l}^{a}=X_{\;j,l}^{a}]=\left(n_{a}-2\right)+2P\left(X_{i}=X_{j}\;|\;l\right).$$

This identity follows from:

$$\begin{array}{rl} \sum_{a}\left[X_{i,l}^{a}=X_{\;j,l}^{a}\right] & =\sum_{a}\left(P(X_{i}=X_{j}=a\;|\;l)+P(X_{i}\neq a,X_{j}\neq a\;|\;l)\right)\\ & =P(X_{i}=X_{j}\;|\;l)+\sum_{a}(1-P(X_{i}=a,X_{j}\neq a\;|\;l)\\ & \;-P(X_{i}\neq a,X_{j}=a\;|\;l)-P(X_{i}=X_{j}=a\;|\;l))\\ & =n_{a}-\sum_{a}\left(P(X_{i}=a\;|\;l)-P(X_{i}=X_{j}=a\;|\;l)\right)\\ & \;-\sum_{a}\left(P(X_{j}=a\;|\;l)-P(X_{i}=X_{j}=a\;|\;l)\right)\\ & =n_{a}-2+2P(X_{i}=X_{j}\;|\;l). \end{array}$$

Then, using Lemma C1 we can write (4) as:

$$\begin{array}{rl} C_{ij} & =\sum_{a}\frac{1}{2}\left(P(X_{i}=X_{j}=a)-P(X_{i}=X_{U}=a\right)\\ & \;-P(X_{j}=X_{V}=a)+P(X_{U}=X_{V}=a))\\ & =\frac{1}{2n_{l}}\sum_{l=1}^{n_{l}}\sum_{a}\frac{1}{2}\frac{1}{n_{i}^{2}}\sum_{u=1}^{n_{i}}\sum_{v=1}^{n_{i}}\left([X_{i,l}^{a}=X_{\;j,l}^{a}]-[X_{i,l}^{a}=X_{u,l}^{a}\right]\\ & \;-[X_{\;j,l}^{a}=X_{v,l}^{a}]+[X_{u,l}^{a}=X_{v,l}^{a}])\\ & =\frac{1}{2n_{l}}\sum_{l=1}^{n_{l}}\sum_{a}\frac{1}{n_{i}^{2}}\sum_{u=1}^{n_{i}}\sum_{v=1}^{n_{i}}\left(X_{i,l}^{a}-X_{u,l}^{a}\right)\left(X_{\;j,l}^{a}-X_{v,l}^{a}\right).\\ & =\frac{1}{2n_{l}}\sum_{l=1}^{n_{l}}\sum_{a}(X_{i,l}^{a}-p_{l}^{a})(X_{\;j,l}^{a}-p_{l}^{a}) \end{array}$$

Note that again this expression agrees with equation (1), after dividing the equation by $n_{l}$.

## Appendix D: Covariance between uncentered traits

Without centering to the sample mean, the covariance between the traits of individuals *i* and *j* is:

(D1 )
$$C\text{ov}\left[Z(i),Z(\;j)\right]=\sigma^{2}\sum_{\ell =1}^{n_{L}}p(i,\ell )p(\;j,\ell ).$$

Note that this expression depends on the choice of ancestral allele. This seems undesirable for a measure of relatedness; choosing a point farther back in time as a reference, so that a different allele is “ancestral” and the derived allele is likely fixed, should not affect relatedness within the population. It does affect the relatedness calculated above because this is, implicitly, a model of trait variation *relative* to a hypothetical individual whose genotype is composed entirely of ancestral alleles. A common approach to resolve this, as we do in the main paper, is to *center* the traits, which takes the mean of some individuals as reference, rather than a hypothetical ancestor. When using pedigree data, these reference individuals are founders of the pedigree (Wright 1922), while when using genotype data these reference individuals are the genotyped individuals (VanRaden 2008).

Similarly, if the branch GRM is defined without subtracting sample means, then covariance depends on the presence of branches above the roots of marginal trees. Such branches are inherited by everyone, and in principle there should perhaps be a branch from each root stretching back infinitely into the past, but such branches are usually omitted. Centering removes their effect.

## Appendix E: Branch PCA on a single chromosome

Figure E1 shows the same analysis as Fig. 1, but computed using a single chromosome: Figs. E1D-F are from the pedigree and so identical, but Figs. E1A-C are computed from simulations of only a single chromosome.

## Appendix F: Comparison of branch GRM and genotype GRM

Figure F1 compares the branch GRM and genotype GRM calculated from the same ARG, illustrating very strong similarity between the two. The correlation between the two GRMs is 0.9998219.

## Appendix G: Benchmarking branch GRM computations

We compared ts.genetic_relatedness_matrix for computing the branch GRM to the implementation in the eGRM package (Fan et al. 2022). See section Branch GRM for details of the benchmarking experiment. Figure G1 demonstrates that the tskit implementation consistently outperforms the eGRM implementation.

## Appendix H: Proof of correctness of algorithm V

Here we prove that when Algorithm **V** completes, v(s) is equal to the $s^{\mathrm{th}}$ entry of Bw, as defined in (15). In fact, after each step in the algorithm (i.e. after each addition or removal of an edge), it is true that for *every* node *n*, the sum of everything above that node is equal to the weighted sum of covariances for that node including everything up to that point in the genome. In other words, for every *n*:

(H1 )
$$\;S_{T}(n)\;:\;=\sum_{r\geq_{T}n}v(r)+z(r)w(r)$$

(H2 )
$$\;\;=\sum_{h\;:\;h\leq k}(b_{h}-b_{h-1})\sum_{r\;:\;n\leq_{T_{h}}r}\ell_{T_{h}}(r)\sum_{t\;:\;t\leq_{T_{h}}r}w_{t}$$

where *T* is the current tree. This statement reduces to our claim that the algorithm is correct because the final tree is an “empty” tree with no edges, so at the end of the algorithm, the left-hand side is simply v(n)+z(n). This is again v(n) because z(n) is zero due to $x(n)=b_{K}$. The right-hand side is equal to equation (15) when *n* is a sample node.

Each time we add an edge with child *c* and parent *p* to the tree (step **V2**), we add the value of w(n) to *p* and all nodes above *p* in the tree; when removing edges we subtract (step **V1**). Since w(n) is initialized so that each sample *s* carries $w_{s}$, this ensures that $w(n)=\sum_{s\leq n}w_{s}$ at all times [as in Kelleher et al. 2016; Ralph et al. 2020].

We prove that equation (H1) is always true by induction. At the first (empty) tree, this is certainly true, as both sides are equal to zero. We now consider **Step V3**. Tree *T* and the bookkeeping variables *v*, *w* and *x* are left constant. Advancing the position from *k* to k+1 only changes $z(s)=\ell_{T}(s)(b_{k}-x(s))$ to $z^{\prime }(s)=\ell_{T^{\prime }}(s)(b_{k+1}-x(s))$. Therefore, the appended value to the left-hand side is

(H3 )
$$\sum_{r\;:\;n\leq_{T}r}z^{\prime }(r)w(r)-z(r)w(r)=(b_{k+1}-b_{k})\sum_{r\;:\;n\leq_{T}r}\ell_{T}(r)w(r)$$

which is also equal to the added value to the right-hand side. Note that this is the only step that changes the value of equation (H1).

**Step V1** and **V2** leave the value of both sides unchanged. Without the loss of generality, we prove this for **V2**. Suppose that we added edge (c,p) where *c* and *p* are the child and the parent nodes of the edge, respectively. We can divide the nodes of N into four categories as (1) the child node *c*, (2) nodes below *c*, (3) the parent node *p* and nodes above *p*, and (4) all other nodes. The insertion changes the values of the intermediate arrays to $v^{\prime }$, $w^{\prime }$, $z^{\prime }$, and $x^{\prime }$ following **V2**. We denote the new tree resulting from the insertion as $T^{\prime }$.

Observe that the difference in the left-hand side after V2 is

(H4 )
$$\begin{array}{rl} & \sum_{r\;:\;n\leq_{T^{\prime }}r}v^{\prime }(r)+z^{\prime }(r)w^{\prime }(r)-\sum_{r\;:\;n\leq_{T}r}v(r)+z(r)w(r)\\ & \;=\sum_{r\;:\;n\leq_{T^{\prime }}r,n{≰}_{T}r}v^{\prime }(r)+z^{\prime }(r)w^{\prime }(r)\\ & \;+\sum_{r\;:\;n\leq_{T^{\prime }}r,n\leq_{T}r}v^{\prime }(r)+z^{\prime }(r)w^{\prime }(r)-v(r)-z(r)w(r)\\ & \;-\sum_{r\;:\;n{≰}_{T^{\prime }}r,n\leq_{T}r}v(r)+z(r)w(r)\\ & \;=\sum_{r\;:\;n\leq_{T^{\prime }}r,n{≰}_{T}r}v^{\prime }(r)+z^{\prime }(r)w^{\prime }(r)\\ & \;+\sum_{r\;:\;n\leq_{T}r}v^{\prime }(r)+z^{\prime }(r)w^{\prime }(r)-v(r)-z(r)w(r) \end{array}$$

for node *n*. The last line follows from $\left\{r\;:\;n\leq_{T}r\}\subset \{r\;:\;n\leq_{T^{\prime }}r\right\}$ because $T^{\prime }$ has more edges than *T*. Nodes in each category have distinct values for the former and the latter sum of this equation.

The set $\left\{r\;:\;n\leq_{T^{\prime }}r,n{≰}_{T}r\right\}$ of the first summation is

(H5 )
$$\left\{ \begin{array}{cc} \left\{r\;:\;p\leq_{T^{\prime }}r\right\} & \;n\;\text{in category ( 1)\textbackslash{}; or ( 2) }\\ \emptyset & \;n\;\text{in category ( 3)\textbackslash{}; or ( 4) } \end{array}\right.$$

This is because *p* and the nodes above *p* are the nodes that were previously not ancestors in *T*, but became ancestors of *c* and those below *c* after the addition of the new edge. The set is empty for the nodes in the third and the fourth category because their ancestor nodes are unchanged after edge insertion. Therefore, the first summation is

(H6 )
$$\left\{ \begin{array}{cc} \sum_{r\;:\;p\leq_{T^{\prime }}r}v^{\prime }(r)+z^{\prime }(r)w^{\prime }(r) & \;n\;\text{in category ( 1)\textbackslash{}; or ( 2) }\\ 0 & \;n\;\text{in category ( 3)\textbackslash{}; or ( 4) } \end{array}\right.$$

The summand of the second summation $v^{\prime }(r)+z^{\prime }(r)-v(r)-z(r)$ is

(H7 )
$$\left\{ \begin{array}{cc} v^{\prime }(c)-v(c) & \;r\;\text{in category ( 1) }\\ 0 & \;r\;\text{in category ( 2) , ( 3) , or ( 4) } \end{array}\right.$$

For r=c (*r* is in the first category), it follows from z(c)=0 due to $\ell_{T}(c)=0$ and $z^{\prime }(c)=0$ due to $x^{\prime }(c)=b_{k}$. All the bookkeeping values of the second and the fourth category is unchanged by **V2**, so the summand is trivially zero. When r=p (*r* belongs to the third category), $v^{\prime }(r)=v(r)+z(r)w(r)$ and $z^{\prime }(r)=0$ by the operations v(r)+=z(r)w(r) and $x(r)=b_{k}$ by **V2**. Hence, the second summation is

(H8 )
$$\left\{ \begin{array}{cc} v^{\prime }(c)-v(c) & \;n\;\text{in category ( 1)\textbackslash{}; or ( 2) }\\ 0 & \;n\;\text{in category ( 3)\textbackslash{}; or ( 4) } \end{array}\right.$$

because the set $\left\{r\;:\;n\leq_{T}r\right\}$ contains *c* if and only if *n* belongs to either the third or the fourth category. An expression for $v^{\prime }(c)-v(c)$ comes from the operation v(c)−=v(r) following v(r)+=z(r)w(r) of **V2**

(H9 )
$$v^{\prime }(c)-v(c)=-\sum_{r\;:\;p\leq_{T}r}v(r)+z(r)w(r)$$

Combining the aforementioned results, we see that equation (H4) is

(H10 )
$$\left\{ \begin{array}{cc} \begin{array}{c} \sum_{r\;:\;p\leq_{T^{\prime }}r}v^{\prime }(r)+z^{\prime }(r)w^{\prime }(r)\\ -\sum_{r\;:\;p\leq_{T}r}v(r)+z(r)w(r) \end{array} & n\in \;(\text{1)\textbackslash{}; or ( 2})\\ 0 & n\in \;(\text{3) \textbackslash{};or ( 4}) \end{array}\right.$$

Both cases reduces to zero because the set of nodes ancestral to *p* are the same in *T* and $T^{\prime }$ ($\left\{r\;:\;p\leq_{T}r\}=\{r\;:\;p\leq_{T^{\prime }}r\right\}$), and $v^{\prime }(r)=v(r)+z(r)w(r)$ for these nodes due to **V2** and $z^{\prime }(r)=0$.

The right-hand side remains the same because the operation changes nothing in its expression: it’s the working sum until the previous local tree that it does not contain any of the components of the current tree that is being modified. This completes the proof.

## Appendix I: Computational scaling of the branch GRM-vector product algorithm

Figure I1 plots the time taken to compute the branch GRM-vector product against subsets of a large simulated ARG (Anderson-Trocmé et al. 2023) of chromosome 21.
